# Control of yeast retrotransposons mediated through nucleoporin evolution

**DOI:** 10.1371/journal.pgen.1007325

**Published:** 2018-04-25

**Authors:** Paul A. Rowley, Kurt Patterson, Suzanne B. Sandmeyer, Sara L. Sawyer

**Affiliations:** 1 BioFrontiers Institute, Department of Molecular Cellular and Developmental Biology, University of Colorado Boulder, Boulder, CO, United States of America; 2 Department of Biological Sciences, University of Idaho, Moscow, ID, United States of America; 3 Department of Biological Chemistry, School of Medicine, University of California, Irvine, Irvine, CA, United States of America; Cornell University, UNITED STATES

## Abstract

Yeasts serve as hosts to several types of genetic parasites. Few studies have addressed the evolutionary trajectory of yeast genes that control the stable co-existence of these parasites with their host cell. In *Saccharomyces* yeasts, the retrovirus-like Ty retrotransposons must access the nucleus. We show that several genes encoding components of the yeast nuclear pore complex have experienced natural selection for substitutions that change the encoded protein sequence. By replacing these *S*. *cerevisiae* genes with orthologs from other *Saccharomyces* species, we discovered that natural sequence changes have affected the mobility of Ty retrotransposons. Specifically, changing the genetic sequence of *NUP84* or *NUP82* to match that of other *Saccharomyces* species alters the mobility of *S*. *cerevisiae* Ty1 and Ty3. Importantly, all tested housekeeping functions of *NUP84* and *NUP82* remained equivalent across species. Signatures of natural selection, resulting in altered interactions with viruses and parasitic genetic elements, are common in host defense proteins. Yet, few instances have been documented in essential housekeeping proteins. The nuclear pore complex is the gatekeeper of the nucleus. This study shows how the evolution of this large, ubiquitous eukaryotic complex can alter the replication of a molecular parasite, but concurrently maintain essential host functionalities regarding nucleocytoplasmic trafficking.

## Author summary

Genomes are the blueprint of life, but they are also plagued by parasites. Genomic parasites are elements like transposons, which are strings of genetic sequence with the capability of propagating through genomes. It is interesting to consider how organisms evolve to protect their genomes from the unchecked propagation of transposons. Here, we show that the genes encoding certain nuclear pore components in yeast have evolved to alter the mobility of Ty retrotransposons. We investigate the evolutionary and functional outcomes of this relationship, and speculate on how such a conserved structure as the nuclear pore could afford to change protein sequence while still performing its conserved functions.

## Introduction

The presence of Ty retrotransposons (Tys) in all species of *Saccharomyces* yeasts suggest that they have likely been coevolving together for about 20 million years [1,2]. Because Tys are strictly intracellular parasites, both the host (yeast) and Tys are aligned in benefitting from a controlled, sustained relationship that does not place the host at an evolutionary disadvantage [3]. This might even be thought of as a symbiotic relationship because, unlike most pathogenic viruses of higher eukaryotes, Tys are a force for genetic plasticity, driving adaptive changes within the yeast genome in response to changes in environmental conditions [4]. For this reason, it is thought that both Tys and the host genome have evolved mechanisms to attenuate unchecked Ty replication that would place an excessive burden on the host cell [3,5–10]. Thus, yeasts have likely experienced selection to control genetic parasites [11,12]. In turn, Tys may counter-adapt to evade host control strategies, or may adapt to modulate their own pathogenicity. Regardless of whether a Ty is thought of as a symbiont, or a “tamed” parasite, one can imagine that the host-parasite relationship must be finely tuned within each yeast species, with different evolutionary strategies emerging over evolutionary time (in both yeast and Ty) to control Ty replication.

There are many examples of genetic parasites, including viruses and transposable elements, that must access the nucleus of a host cell in order to replicate. Thus, the nuclear envelope represents a major barrier to these parasites in their eukaryotic hosts [13–15]. The movement of large macromolecules between the cytoplasm and the nucleus occurs though the nuclear pore complex. The nuclear pore complex is composed of multiple copies of approximately 30 different proteins, referred to as nucleoporins, and is conserved between yeast and higher eukaryotic species, including humans [16–22]. Transport receptors, called karyopherins, facilitate the transport of cellular cargo through the nuclear pore [20,23]. Genetic parasites interact with a wide variety of nucleoporins and karyopherins to facilitate the nucleocytoplasmic transport of their proteins and complexes, or to re-localize useful or antagonistic host proteins [24–33].

**Fig 1:**
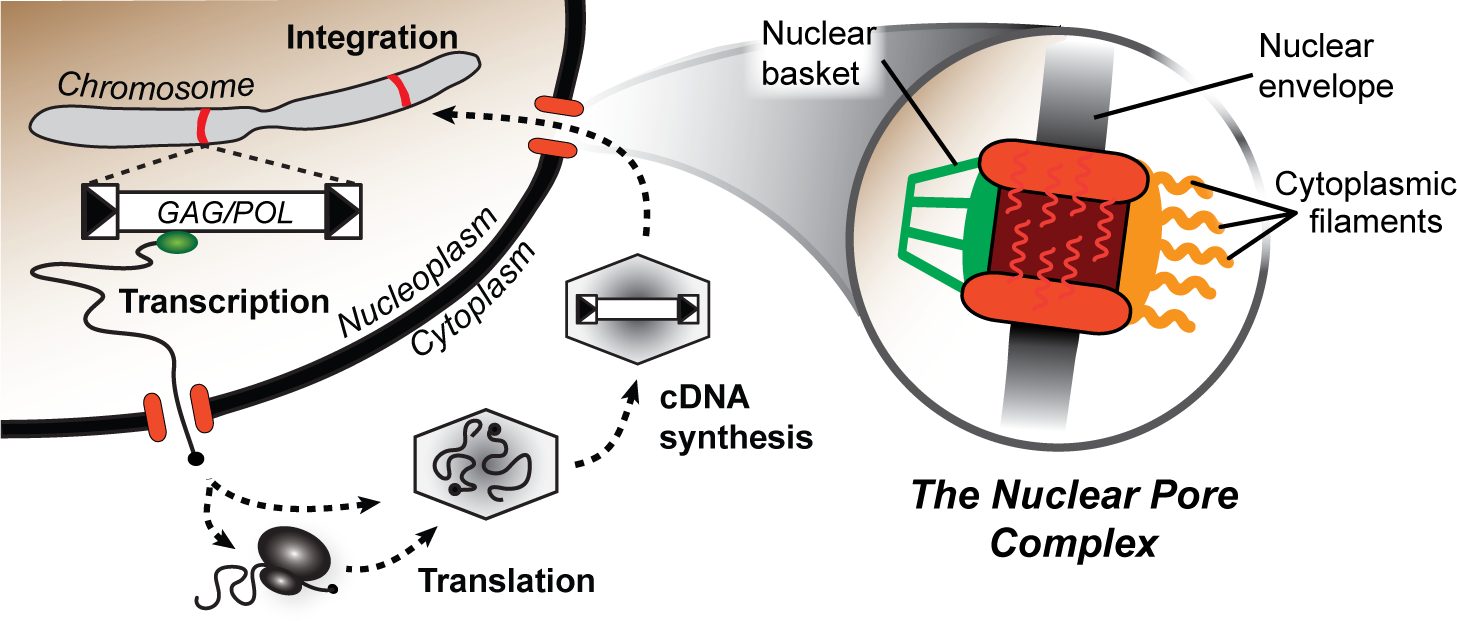
The nuclear pore complex is important for Ty retrotransposition. *Left*. A generic schematic of the lifecycle of a Ty. Chromosomal copies of Ty, found in the yeast genome, produce full-length RNA transcripts that are exported from the nucleus. These transcripts are translated and also packaged within virus-like particles within the cytoplasm. Packaged RNAs are reverse transcribed into cDNA that is transported into the nucleus via the nuclear pore complex. The Ty integrase mediates insertion of the cDNA into the host genome at a new location (red stripes on the chromosome). *Right*. Simplified representation of the nuclear pore complex embedded in the nuclear envelope and sliced along its vertical axis. Filaments rich in phenylalanine and glycine (FG) radiate into the nucleoplasm, cytoplasm, and within the nuclear pore itself.

*Saccharomyces* yeasts are eukaryotes that play host to a variety of DNA plasmids, single-stranded RNA viruses (from the family *Narnaviridae*), double stranded RNA viruses (from the family *Totiviridae*), and Ty retrotransposons [34–36]. Of these viruses and virus-like elements, only Tys transit through the nuclear pore complex. There are five families of Tys in *S*. *cerevisiae*, Ty1 to Ty5, and all have an analogous lifecycle to retroviruses [37–39]. Tys have intracellular lifecycles (Fig 1), but can be transmitted to new hosts via yeast mating [40]. The Ty lifecycle involves the movement of Ty components between the cytoplasm and the nucleus every replication cycle via the nuclear pore complex. Ty3 virus-like particles and proteins have been observed to cluster at the nuclear envelope and the cytoplasmic face of the nuclear pore complex [25]. Multiple Ty3 proteins (Gag3, p27 and CA) interact directly with nucleoporins, and the Ty1 and Ty3 integrase (IN) proteins contain nuclear localization signals [25,41–44]. Together, these factors presumably direct the nuclear ingress of Ty cDNA and associated proteins. After nuclear entry, integrase catalyzes the insertion of Ty cDNA into the host genome [45,46]. Tys must also exit the nucleus. Ty1 RNAs, after transcription in the nucleus, are thought to be stabilized and chaperoned from the nucleus by the Gag protein [47].

**Fig 2:**
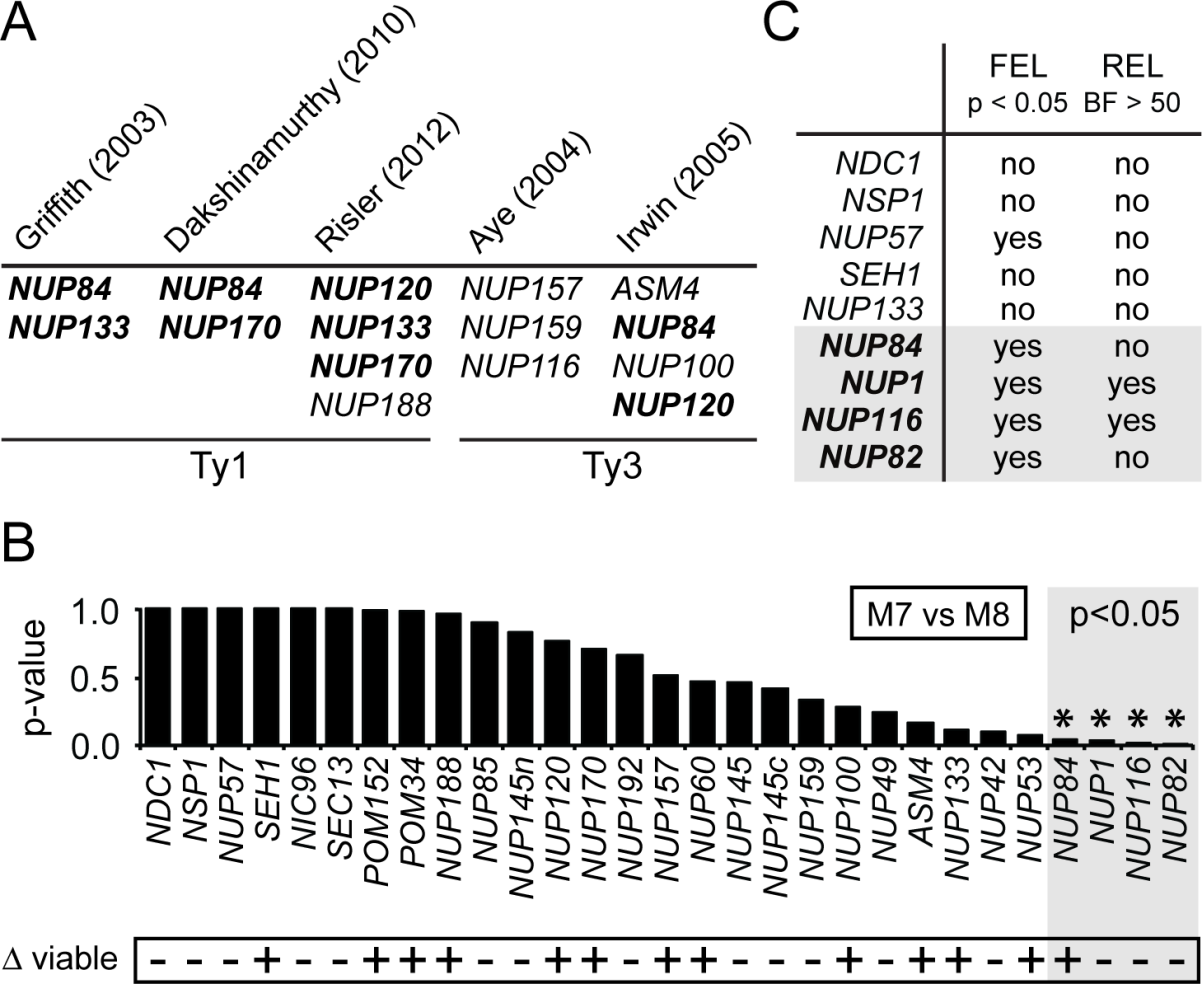
Nucleoporins are evolving rapidly in *Saccharomyces* yeasts. (A) Results of published high-throughput genetic screens for host factors affecting Ty mobility [51,52,55–57]. Only nucleoporin genes found in these screens are summarized, where disruption of the indicated gene altered Ty3 or Ty1 mobility. Bold text indicates genes found in more than one screen. (B) Results from PAML analysis surveying nucleoporin genes for codons with elevated evolutionary rate (dN/dS ≥1). Here, alignments were fit to a codon model of conservative evolution (M7) and a codon model allowing for codons with an elevated evolutionary rate (M8). M7 was rejected in favor of M8 for four nucleoporins (p<0.05): *NUP84*, *NUP1*, *NUP116* and *NUP82*. Along the bottom is summarized whether yeast with a deletion of each of these genes is viable, taken from the *Saccharomyces* genome database. (C) Extended evolutionary analysis of selected nucleoporins using two additional tests for positive selection (FEL and REL) [68]. “Yes” indicates that codons with dN/dS>1 were detected in this gene by the indicated test, with a p-value (p) < 0.05, or Bayes factor (BF) > 50.

Because the lifecycle of Tys involves trafficking in and out of the nucleus, we investigated the hypothesis that nucleoporins might experience evolutionary pressure to control Ty nucleocytoplasmic transport. While evolution of host immune strategies is common [48–50], evolved resistances have not been extensively documented in large, essential cellular assemblages, such as the nuclear pore complex. Seven published high-throughput screens have been conducted in order to identify genes important for the replication of Ty1 (five studies [51–55]) or Ty3 (two studies [56,57]). Among these studies, ten nucleoporins (Fig 2A) and four karyopherins (S1 Fig) were identified as important for Ty replication. Several genes were identified in multiple screens, as represented in Figs 2A and S1. Interestingly, the knockout of some nuclear pore-related genes has been noted to reduce Ty mobility, while the knockout of others increases it [58]. One possible interpretation of this intriguing pattern is that there is a highly evolved relationship between yeasts and Tys. In some cases, Tys are successfully exploiting a nuclear pore protein for import/export. Knockout of such genes would reduce Ty mobility. In other cases the host may have evolved to reduce Ty transport, for instance by evolving a nuclear pore protein that binds but does not transit Ty componentry, or that binds Ty componentry and mis-localizes it. Deleting these genes would increase Ty mobility. There are likely to be additional nuclear pore complex-related genes, beyond those shown in Figs 2A and S1, that are involved in Ty replication. This is because genes essential to yeast viability are usually underrepresented in such screens, given that gene knockouts of these genes are inviable.

To further explore the idea of evolved control of Tys, we looked at the evolutionary history of all known *Saccharomyces* nucleoporin genes, and found that 26 of 30 nucleoporins have changed very little during *Saccharomyces* speciation and are evolving under purifying selection. However, four nucleoporins are evolving rapidly in a manner consistent with positive selection (*NUP1*, *NUP82*, *NUP84*, and *NUP116)*. We wished to explore how the high level of sequence divergence in these proteins between species affects Ty control. For *NUP82* and *NUP84*, we engineered *S*. *cerevisiae* strains to express orthologs from other yeast species and then assayed the replicative success of different families of Tys within these otherwise isogenic yeast strains. We found that species-specific evolutionary differences in these nucleoporins affected the replication of either Ty1, Ty3, or both Ty families. *NUP84* appears to have experienced selection primarily to control Ty1, while *NUP82* has experienced selection primarily to control Ty3. Moreover, Nup82p and Nup84p are integral to the nuclear pore complex structure and are required for its functionality [59,60]. We find that adaptive changes in *NUP82* and *NUP84* affect Ty replication, yet have accumulated under the constraints of strict conservation of nucleoporin host functions during *Saccharomyces* speciation.

## Results

### *NUP82* and *NUP84* have accumulated elevated levels of non-synonymous substitutions

We first set out to determine which nuclear pore complex-related genes might be important in the evolved control of Tys. Obviously, genes that have remained unchanged over the speciation of *Saccharomyces* yeast would be unlikely to fall into this class. Instead, as a screening tool we sought genes that have diverged significantly in sequence from one yeast species to the next. We are particularly interested in genes with evidence for natural selection underlying these sequence changes, rather than genes that have diverged in sequence simply by the forces of random genetic drift. Natural selection can be detected in genes as follows. Typically, selection operates on non-synonymous substitutions (changing the encoded amino acid) more significantly than on non-synonymous mutations (silent, not changing the encoded amino acids). Gene regions that have experienced repeated rounds of natural selection in favor of protein-altering mutation therefore exhibit a characteristic inflation of the rate of non-synonymous (dN) DNA substitutions compared to synonymous (dS) substitutions (denoted by dN/dS > 1) [61]. Because non-synonymous mutations occur more often than synonymous mutations by random chance, computational models have been developed that use statistical frameworks to account for these unequal substitution rates [62–64]. The mode of evolution that we are seeking (dN/dS > 1) is considered to be somewhat rare in eukaryotic genes. Instead, most genes experience purifying selection (dN/dS < 1), where protein sequence is conserved over evolutionary time due to the important and complex roles that most proteins play in cellular homeostasis.

We examined the evolution of 29 yeast genes encoding nucleoporins and 22 genes encoding karyopherins for evidence of codons with dN/dS > 1. For each gene, we gathered nucleotide sequences from six divergent *Saccharomyces* species (*S*. *cerevisiae*, *S*. *paradoxus*, *S*. *mikatae*, *S*. *kudriavzevii*, *S*. *arboricolus* and *S*. *bayanus*) [65–67]. Next, we constructed DNA alignments of the various genes and fit these to two different models of codon evolution using the Phylogenetic Analysis by Maximum Likelihood (PAML) package [64]. Evolutionary model M7 was used as our null model and assumes that all codons within a gene are evolving conservatively (dN/dS > 1 not allowed), whereas model M8 allows for some codons to exhibit an elevated evolutionary rate (dN/dS ≥ 1). Model M7 was rejected in favor of M8 (p<0.05) for four nucleoporin genes: *NUP84*, *NUP1*, *NUP116* and *NUP82* (Fig 2B). The null model was not rejected for any karyopherins (S1 Fig). Interestingly, one of these nucleoporin genes, *NUP84*, is also the only nuclear pore-related gene found in three different knockout screens as important for Ty mobility (Fig 2A). *NUP133*, *NUP120*, and *NUP170*, which were found in two independent genetic screens (Fig 2A) did not pass the threshold of significance (p>0.05; Fig 2B), and so were not investigated further. The remaining three nucleoporin genes under positive selection (*NUP1*, *NUP116* and *NUP82*) are essential genes within *S*. *cerevisiae* (Fig 2B, bottom), and of these, only *NUP116* has been directly tested and demonstrated to be involved with Ty replication [25].

Various statistical tests have been designed to detect positive selection, all of which take different approaches to modeling the rates of nonsynonymous and synonymous changes that have occurred in a given gene alignment [69]. We next evaluated *NUP84*, *NUP1*, *NUP116*, and *NUP82* with additional tests for positive selection, FEL and REL [68]. We found that all four nucleoporin genes showed evidence of positive selection using at least one of these additional tests (Fig 2C). Furthermore, three of these genes (*NUP1*, *NUP82*, and *NUP116)* were previously identified as evolving rapidly in a whole genome evolutionary study of five *Saccharomyces* yeast species performed by Scannell *et al*. [67]. In contrast, *NUP133* and four other nucleoporins with the least support for rejection of the M7 null model (*NDC1*, *NSP1*, *NUP57* and *SEH1*; Fig 2B), passed zero or only one of these tests (Fig 2C). We next turned to functionally testing the biological relevance of the observed evolutionary signatures identified within nuclear pore complex-related genes.

### A novel GFP reporter of Ty mobility

**Fig 3:**
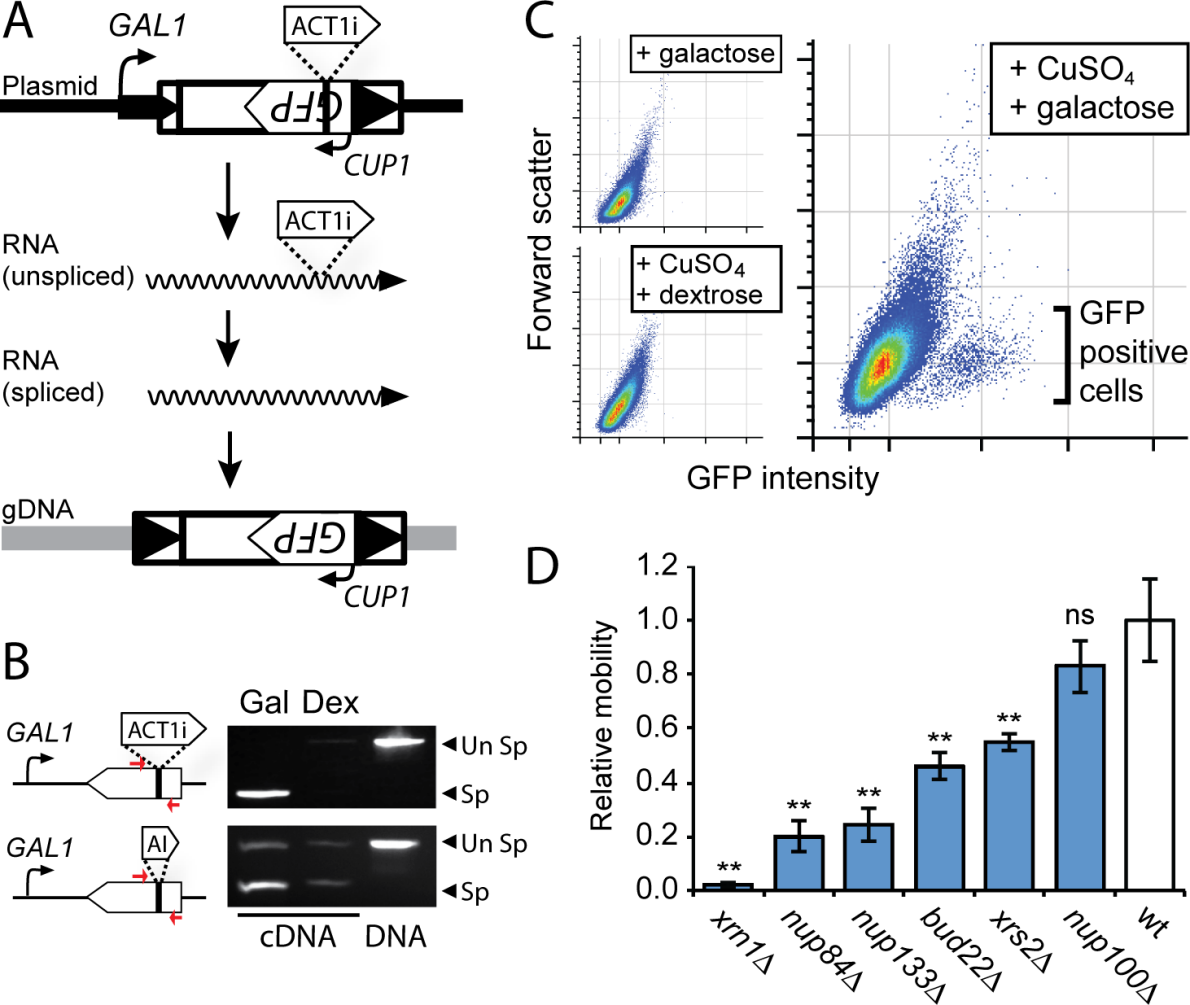
A novel GFP-based reporter of Ty1 mobility. (A) An overview of the GFP-tagged Ty1 plasmid. Ty1 transcription is induced by activation of the *GAL1* promoter that produces a long Ty1 transcript including an internal *GFP* gene and an *ACT1* intron (ACT1i). The spliced transcript has the ACT1i removed, which then provides a template for Ty1 protein production and reverse transcription. Ty1 cDNA is imported into the nucleus and integrated into the *S*. *cerevisiae* genome. The *GFP* gene is then induced from the *CUP1* promoter by CuSO_4_ to report successful integration events. (B) RT-PCR was used to assess splicing of RNA with ACT1i versus an artificial intron (AI) within the *GFP* gene (primer positions marked by red arrows). Spliced RNA transcripts (Sp) were mainly detected upon induction of the transcription by the *GAL1* promoter using galactose (Gal). Growth on dextrose (Dex) inhibits the *GAL1* promoter and the production of RNA transcripts. Plasmid DNA was used as a positive control to allow the PCR amplification across intron-containing *GFP*. “Un Sp” indicated the detection of unspliced RNAs. (C) Flow cytometry analysis shows that *GFP* is only expressed under conditions of galactose induction of Ty1 expression followed by CuSO_4_ induction of *GFP*. (D) The effect of six different gene deletions on Ty1 mobility, relative to wild-type *S*. *cerevisiae*. The relative mobility was measured as a percent of GFP positive cells after induction of the Ty1-GFP reporter, and was repeated independently, three times (error bars: standard error, n>3; **Tukey–Kramer method, p<0.05). All values are normalized to wildtype.

We first built a quantitative, GFP-based assay system for Ty mobility, which is a variation of a previous assay used in this field [70]. In this system, a plasmid-mounted Ty1 genome from *Saccharomyces cerevisiae* was encoded on the Watson (sense) strand, and was engineered to contain an internal *GFP* gene on the Crick (anti-sense relative to the transcript) strand of the DNA (Fig 3A). To prevent its expression directly from the plasmid vector, the *GFP* gene was engineered to contain an antisense intron (on the Watson strand). Thus, only after the full-length Ty1-GFP transcript has been spliced, reverse transcribed, and integrated into the *S*. *cerevisiae* genome can the *GFP* gene be expressed. *GFP* expression is regulated by the inducible copper-sensitive *CUP1* promoter (Fig 3A). Experiments were performed with two different introns within the *GFP* gene in order to determine which was more efficiently spliced from the transcript produced. The more efficient splicing occurred using the *S*. *cerevisiae ACT1* intron (ACT1i) (Fig 3B). GFP-positive cells were only detected by flow cytometry after galactose was added to the growth medium to initiate Ty1 transcription, and subsequent addition of CuSO_4_ to induce expression of the *GFP* reporter (Fig 3C). We tested our Ty1 mobility reporter in isogenic strains deleted for five genes known to be important for efficient Ty1 mobility: BY4741 *xrn1*Δ, *nup84*Δ, *nup133*Δ, *bud22*Δ, and *xrs2*Δ [51,52,55]. Indeed, we see a significant decrease in Ty1 mobility in each deletion strain compared to the wild-type BY4741 background (Fig 3D). As a control, we show that a strain deleted for *NUP100*, which is important for Ty3 mobility [56], but not known to be important for Ty1, supports a level of mobility that is not significantly different from that of a wild-type strain (Fig 3D).

### *NUP84* evolution modulates Ty1 mobility within *S*. *cerevisiae*

**Fig 4:**
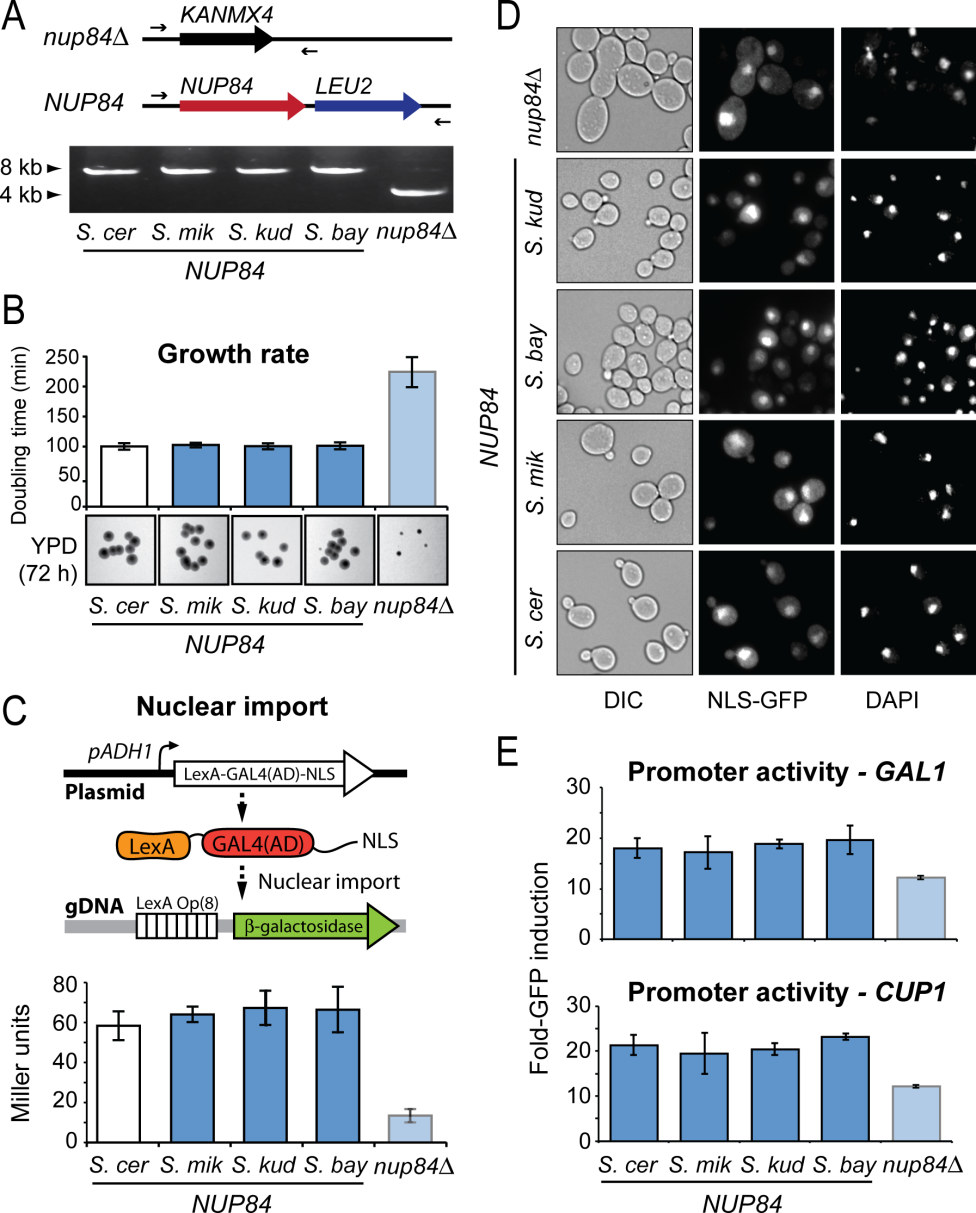
The housekeeping functions of *NUP84* are conserved across divergent *Saccharomyces* species. (A) *Top*. A schematic representation of the *NUP84* locus within *S*. *cerevisiae* engineered to either lack *NUP84* (*nup84*Δ) or to express heterospecific *NUP84* from *S*. *cerevisiae* (*S*. *cer*), *S*. *mikatae* (*S*. *mik*), *S*. *kudriavzevii* (*S*. *kud*) or *S*. *bayanus* (*S*. *bay*) along with the *LEU2* selectable marker. *Bottom*. Successful genome engineering was confirmed by PCR amplification across the *NUP84* locus to detect the replacement of *KANMX4* with *NUP84-LEU2* (primers marked as arrows). (B) The doubling time of *NUP84*-complemented strains in a liquid YPD medium compared to *nup84*Δ, and colony growth and morphology after 72 h of growth on a solid YPD medium. (C) General nuclear import function was assessed in the presence of heterospecific Nup84p or absence of Nup84p using a LexA-Gal4(AD) reporter protein with a SV40 nuclear localization signal (NLS) [71]. The LexA DNA binding domain and Gal4 activation domain (AD) initiate transcription of the β-galactosidase gene upon successful nuclear import. (D) Nuclear transport was also assessed by the steady-state localization of a GFP reporter protein containing a NLS from *PHO4* [72] and its cellular accumulation relative to a DAPI-stained nucleus within *NUP84* complemented *S*. *cerevisiae*. (E) The effect of *NUP84* complementation or deletion on the ability of *S*. *cerevisiae* to express *GFP* from each of the promoters used in the Ty1 *GFP*-based reporter (*GAL1* (top) or *CUP1* (bottom) promoters), using mean fluorescent intensity (MFI) detected by flow cytometry (error bars: standard error, n>3).

*NUP84* is under positive selection and disruption of the gene affects both Ty1 and Ty3 replication (Fig 2). We wished to test whether the evolution of *NUP84* over yeast speciation has altered interactions with Tys. To test this, we replaced *NUP84* within the *S*. *cerevisiae* genome (*NUP84*^*S*.*cer*^) with *NUP84* from diverse *Saccharomyces* species (*S*. *mikatae*, *S*. *kudriavzevii* and *S*. *bayanus*) as outlined in Fig 4A. These sequences encode Nup84p that are between 88% (*S*. *mikatae*) and 85% (*S*. *bayanus*) identical to the *S*. *cerevisiae* protein. As an isogenic control, we re-complemented the *nup84*Δ strain with *S*. *cerevisiae NUP84*. Chromosomal complementation of *S*. *cerevisiae nup84*Δ with each heterospecific (other species) *NUP84* allele resulted in the restoration of normal growth and cellular morphology (Fig 4B and 4D), normal nuclear import (Fig 4C and 4D), and normal gene expression from the promoters used in our Ty1 GFP-based reporter (Fig 4E).

**Fig 5:**
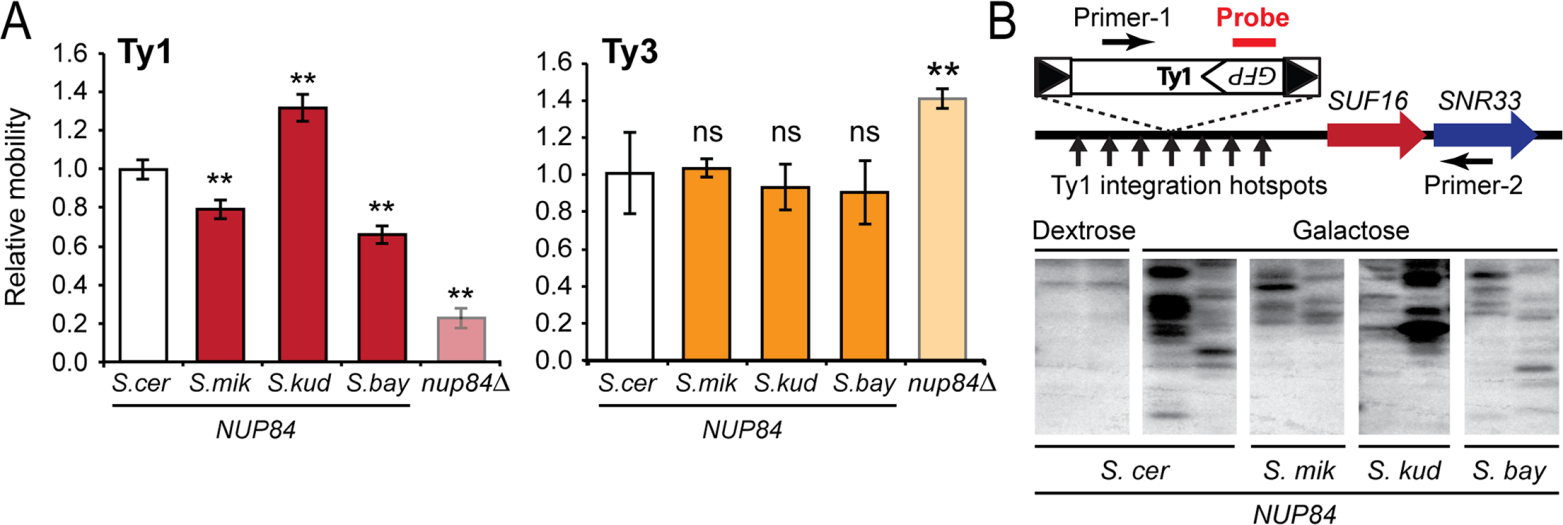
Evolutionary differences between *NUP84* of different *Saccharomyces* species alter levels of Ty1 mobility. (A) Relative mobility of Ty1 and Ty3 within *nup84*Δ or *nup84*Δ complemented with heterospecific *NUP84* from different *Saccharomyces* species. Asterisks designate complemented strains that have significantly different levels of mobility compared to the strain encoding *NUP84* from *S*. *cerevisiae* (Tukey–Kramer method, p<0.05) (error bars: standard error, n>3). (B) Southern blot analysis of Ty1 integration in two independent clones upstream of the *SUF16* locus, which contains Ty1 integration hotspots in its promoter [73]. PCR products across the *SUF16* locus were run on a gel and then probed with a radiolabeled DNA probe specific to *GFP* in order to detect integration events.

The null strain, and each of the four strains expressing wildtype or heterospecific *NUP84*, were transformed with the Ty1 *GFP* reporter described above. Relative to *nup84*Δ, cells complemented with *NUP84*^*S*.*cer*^ increased Ty1 mobility approximately 5-fold (Fig 5A). There were highly significant differences in the levels of Ty mobility among strains encoding heterospecific *NUP84* (one-way ANOVA, p = 8.2 x 10^−8^), and levels of Ty1 mobility were significantly different in strains containing *NUP84*^*S*.*mik*^, *NUP84*^*S*.*kud*^, and *NUP84*^*S*.*bay*^ when compared to *NUP84*^*S*.*cer*^ (Tukey–Kramer method, p<0.05) (Fig 5A). We found that replacement of *NUP84*^*S*.*cer*^ with *NUP84*^*S*.*kud*^ increased Ty1 mobility by 32%, whereas *NUP84*^*S*.*mik*^ and *NUP84*^*S*.*bay*^ both significantly decreased mobility by 21% and 35%, respectively. To verify the observed differences in control of Ty1 mobility, we used Southern blotting to detect Ty1 integrations in the 5’ UTR of the *SUF16* locus, as previously described [73]. We used our *GFP* reporter assay to initiate Ty1 mobility, with Ty1 genomic integrations only detected after induction by galactose (Fig 5B). Similar to our *GFP* reporter assay, fewer integrations were detected within strains encoding *NUP84*^*S*.*mik*^ and *NUP84*^*S*.*bay*^ compared to *NUP84*^*S*.*cer*^. *NUP84*^*S*.*cer*^ and *NUP84*^*S*.*kud*^ had comparable levels of genomic integrations (Fig 5B). To further ensure the generality of our findings, we also measured Ty1 mobility on a single-copy plasmid under both high and low expression conditions. We used the integration of a *HIS3* gene as a marker of successful Ty1 mobilization by assaying the appearance of colonies able to grow on a histidine deficient medium [70]. Again, we were able to observe that heterospecific substitutions were able to alter Ty mobility, even with a 6–9 fold decrease in overall mobility from a single copy plasmid (S2 Fig). Results were broadly consistent between the three assays, with the exception of the heterospecific swap of *S*. *kudriavzevii NUP84*, which reduced Ty1 mobility in the low copy assay (S2 Fig).

These data show that evolutionary differences between *NUP84* of different *Saccharomyces* species modulate the efficiency of Ty1 mobility in a species-specific manner, even though all host functions are conserved. Pairing Ty1 from *S*. *cerevisiae* with *NUP84* of other species apparently decouples a finely co-evolved relationship, altering levels of Ty1 mobility. To support this model, we also assayed the impact of *NUP84* evolution on Ty3 replication. We used a galactose inducible Ty3 with a *HIS3* reporter gene and assayed the appearance of colonies able to grow on a histidine deficient medium [70,74–76]. In contrast to Ty1, we found that *nup84*Δ resulted in increased Ty3 mobility, as was previously reported [56]. However, each of the heterospecific *NUP84* genes returned transposition to the lower level with no significant difference in mobility among strains encoding heterospecific *NUP84* (one way ANOVA, p = 0.90) (Fig 5A). Collectively, these data suggest that the co-evolutionary dynamics are specific to *NUP84* and Ty1.

### *NUP82* evolution alters both Ty1 and Ty3 mobility

**Fig 6:**
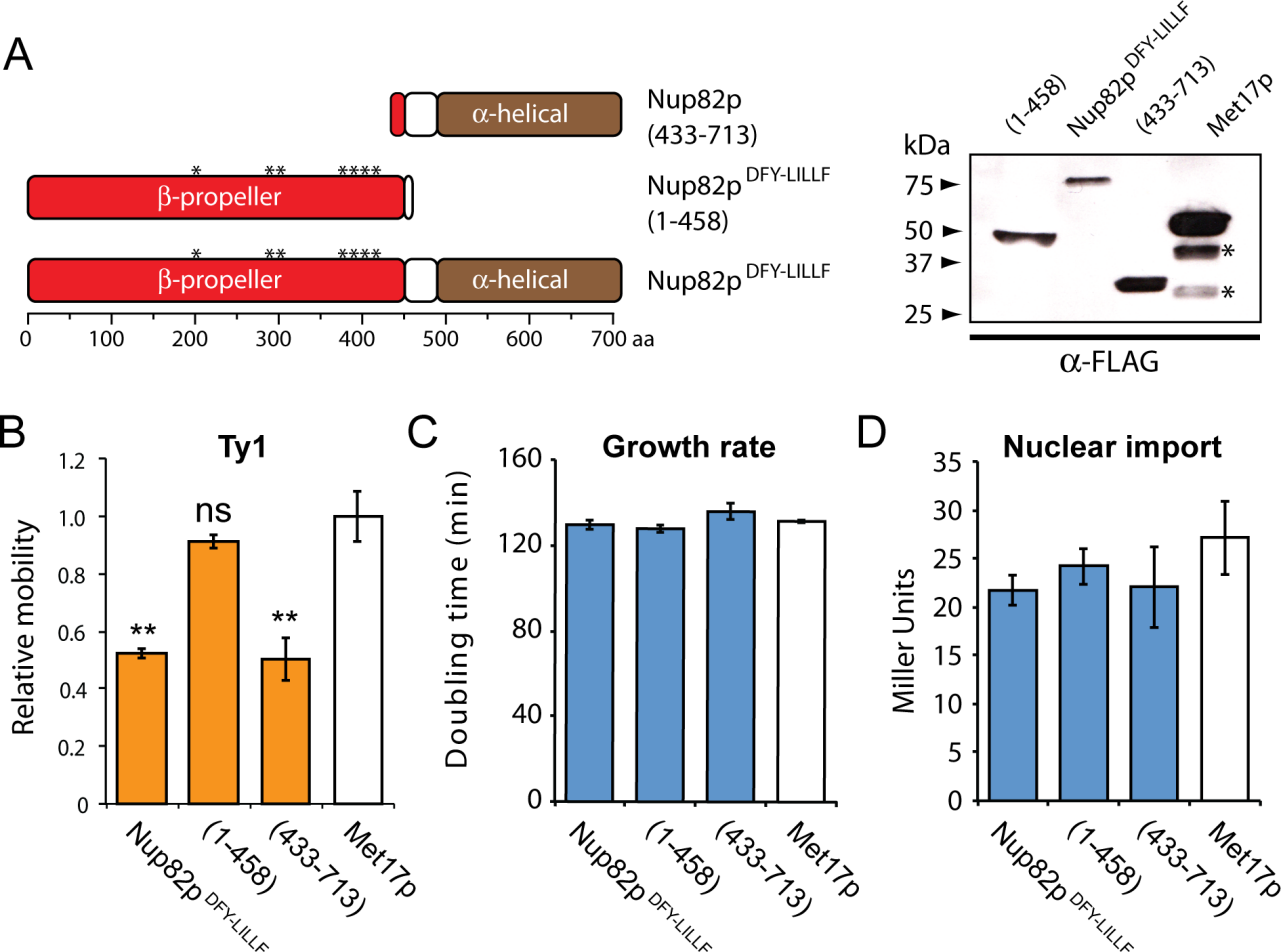
The expression of dominant negative *NUP82* and its impact on Ty1 mobility. (A) *Left*. A linear domain diagram of Nup82p^DFY-LILLF^ and derived deletion mutants [Nup82p(433–713) and Nup82p^DFY-LILLF^(1–458)]. Asterisks mark the mutations that decouple Nup82p from the nuclear pore complex. *Right*. Western blot analysis to detect the expression of FLAG-tagged Nup82p^DFY-LILLF^ and its derivatives, compared to the expression of a control protein (Met17p) in the wild-type background (*Met17p degradation products). The effect of Nup82p^DFY-LILLF^ expression on (B) Ty1 mobility, (C) doubling time in a liquid medium and (D) the nuclear import of the reporter protein LexA-MBP-Gal4(AD), relative to the expression of *MET17* (error bars: standard error, n>3; **Tukey–Kramer method, p<0.05).

Our evolutionary analysis also identified the gene *NUP82* as being the highest scoring nucleoporin in our evolutionary screen (Figs 2B and S1), however no role for *NUP82* has been reported in Ty biology. This could be because *NUP82* is an essential gene and would have eluded detection in genome-wide knockout screens. To investigate whether *NUP82* is involved in Ty replication, a dominant negative approach was adopted. Full- or partial-length portions of *NUP82* were expressed in cells that are otherwise wild type at the *NUP82* locus. These Nup82p constructs included the mutations D204A, F290A, Y295A, L393A, I397A, L402A, L405A and F410A (Nup82p^DFY-LILLF^) that inactivate interaction with other nucleoporins and decouple it from the nuclear pore complex [77] (Fig 6A). Nup82p^DFY-LILLF^ is non-functional as a nucleoporin, therefore we reasoned that it would compete with wild-type Nup82p and have an inhibitory effect on mobility if Ty interacts with Nup82p to transit the nuclear pore. Indeed, the expression of the C-terminal helical domain of Nup82p (residues 433–713) significantly reduced Ty1 mobility, with the N-terminal β-propeller domain (residues 1–458) being dispensable for this effect (Fig 6B). Expression of any of the dominant negative *NUP82* genes did not noticeably affect the growth of *S*. *cerevisiae* (Fig 6C) or general nuclear import (Fig 6D) compared to expression of the control gene *MET17*, which suggests that these proteins are not toxic to *S*. *cerevisiae* and do not disrupt the nuclear pore complex. In summary, this serves as preliminary evidence of a previously uncharacterized role for *NUP82* in Ty1 replication.

**Fig 7:**
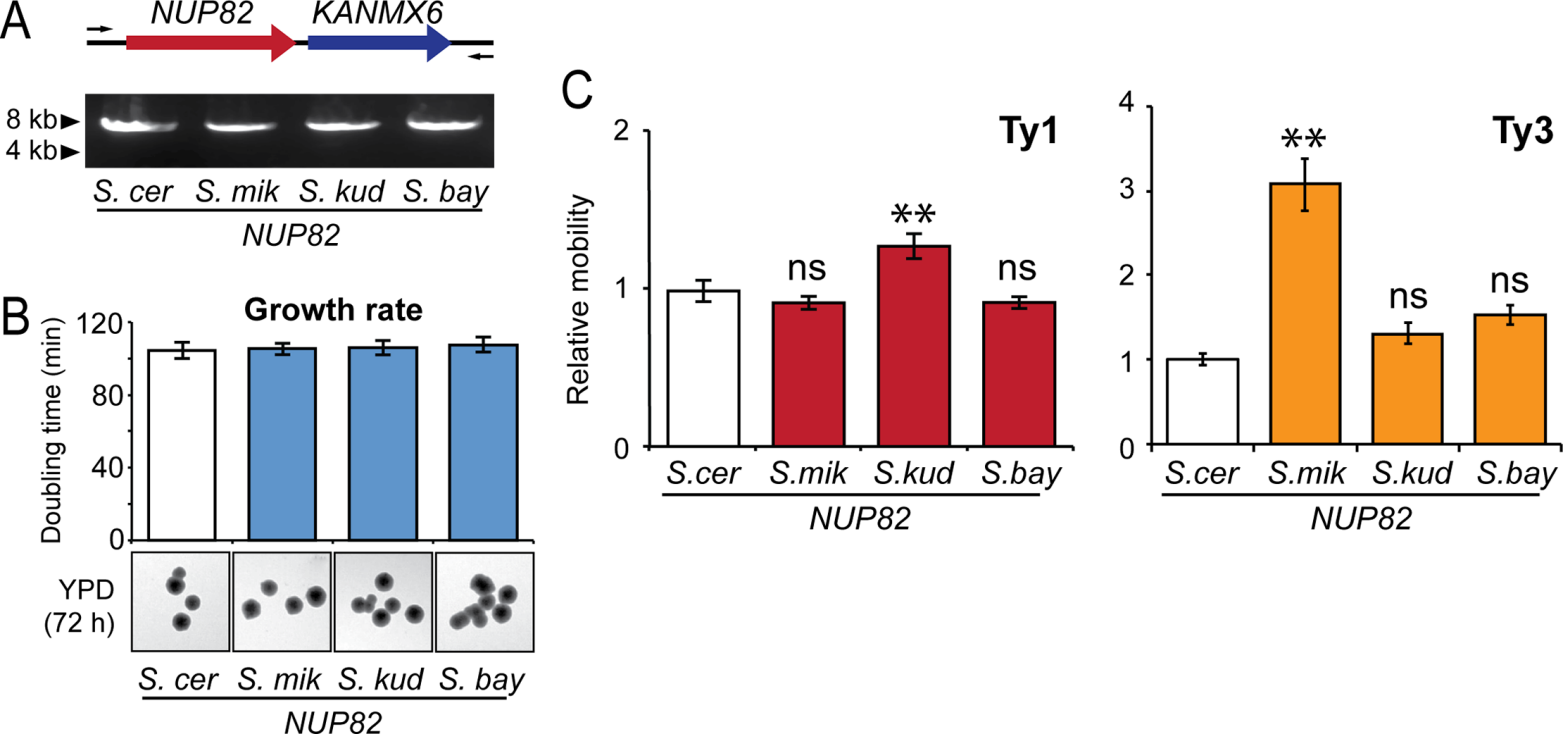
The evolution of *NUP82* and its effect on Ty1 and Ty3 mobility within *S*. *cerevisiae*. (A) A schematic representation of *S*. *cerevisiae* engineered to express *NUP82* from different *Saccharomyces* species. Genome engineering was confirmed by PCR amplification across the *NUP82* locus. (B) The doubling time of *NUP82*-complemented strains in a liquid medium. Colony growth and morphology of the engineered strains was monitored for 72 h on a solid YPD medium (error bars: standard error, n>3). (C) Relative mobility of Ty1 and Ty3 within strains complemented with *NUP82* from different *Saccharomyces* species. Asterisks mark significant differences in Ty mobility compared to the strain encoding *NUP82* from *S*. *cerevisiae* (error bars: standard error, n>3; **Tukey–Kramer method, p<0.05).

Next, in a similar approach to that taken with *NUP84*, *S*. *cerevisiae* was engineered to express *NUP82* from different *Saccharomyces* species to ascertain the impact of *NUP82* evolution on Ty mobility. Due to the essential nature of *NUP82*, we used a *NUP82/nup82*Δ heterozygous diploid strain from the “synthetic genetic array” collection [78] as our starting strain for the genomic replacement of *NUP82*^*S*.*cer*^. A customized SceI restriction endonuclease method was used to improve the efficiency of homologous recombination-based gene replacement (see methods) (Figs 7A and S3). *S*. *cerevisiae* encoding heterospecific *NUP82* have a normal colony morphology, growth rate (Fig 7B), and no difference in *GAL1* and *CUP1* promoter expression (S4 Fig), suggesting that the cells are the same in measurable host functions. We tested the effect of *NUP82* evolution on Ty1 mobility using the GFP fluorescence assay and, in contrast to our studies of *NUP84*, found that Ty mobility levels were similar in strains expressing *NUP82*^*S*.*mik*^ and *NUP82*^*S*.*bay*^ and *NUP82*^*S*.*cer*^, but were significantly higher for strains complemented with *NUP82*^*S*.*kud*^ (Fig 7C). Thus, although *NUP82* may be important for Ty1 mobility (Fig 6), we find that Ty1 seems mostly insensitive to the evolutionary differences between *NUP82* of different species. We next assayed the replication of a Ty3 retrotransposon in the engineered *NUP82* heterospecific strains. *S*. *cerevisiae* expressing *NUP82*^*S*.*mik*^ resulted in a significant >3-fold increase in Ty3 mobility, relative to *NUP82*^*S*.*cer*^ (Tukey–Kramer method, p<0.05) (Fig 7C). These data show that the evolutionary differences within *Saccharomyces NUP82* can impact both Ty1 and Ty3 mobility, but predominantly Ty3. Together, we show that *NUP82* appears to play a previously uncharacterized role in Ty mobility, and that Ty1 and Ty3 are differentially susceptible to evolutionary changes within *NUP82*.

## Discussion

There are many selective pressures driving the evolution of *Saccharomyces* yeasts, including resource competition, sexual selection, and pressure to co-exist with viruses and other genetic parasites [11,12,79–83]. We find signatures of natural selection acting on several nucleoporins, coinciding with previous observations that deletion or disruption of several of these genes can alter Ty mobility. Here, we use a unique approach to demonstrate that the evolutionary changes that have naturally accumulated in yeast nucleoporins can also alter Ty mobility levels, just like laboratory perturbations of these genes are known to do. We successfully replaced *NUP82* and *NUP84*, within the context of the *S*. *cerevisiae* genome, with orthologs from related *Saccharomyces* yeasts, and then demonstrated altered Ty mobility in these isogenic yeast strains. While it is never possible to know for sure what has driven selection within these genes, nucleoporins from different *Saccharomyces* species support variable levels of Ty1 or Ty3 mobility, providing a phenotypic trait on which selection may have been acting. This is similar to our recent observations that the antiviral *XRN1* gene from *Saccharomyces* yeasts has likely co-evolved with totiviruses to control excessive viral replication [11].

It is important to note that, while we have explored nucleoporin evolution, the genetic parasites used in this study have been held constant, with both Ty1 and Ty3 deriving from the *S*. *cerevisiae* lineage. In some cases, orthologs of *NUP82* and *NUP84* resulted in higher levels of *S*. *cerevisiae* Ty mobility, and in other cases, they resulted in lower levels. These patterns are consistent with a model where nucleoporins and Tys are co-evolved in each species. When a Nup82p or Nup84p ortholog is substituted within the *S*. *cerevisiae* nuclear pore, sometimes *S*. *cerevisiae* Tys can exploit it better than it can the *S*. *cerevisiae* version of that protein (possibly by having an increased affinity for the foreign ortholog, which is not evolved to evade *S*. *cerevisiae* Tys). Other times, the *S*. *cerevisiae* Ty is less compatible with this orthologous protein. Ultimately, our data show that the replacement of *S*. *cerevisiae* nucleoporins with heterospecific nucleoporins “decouples” this interaction and leads to either an increase or decrease in Ty mobility, without impacting cellular homeostasis (e.g. nuclear import). It is tempting to try to extrapolate from this study predictions that certain yeast species are better at controlling Ty retrotransposons than others, but this study alone cannot support such conclusions. In this study, we assayed only *S*. *cerevisiae* Tys, but if we assayed Tys from other species, we might expect them to have evolved an optimal interaction with their cognate nuclear pore machinery. Thus is the nature of evolutionary arms race dynamics [48–50].

The exact functions of *NUP82* and *NUP84* during Ty replication remain unclear. Ty nuclear ingress likely involves docking of the virus-like particle to the nuclear periphery by interaction with nucleoporins [25]. The known positioning of Nup82p and Nup84p at the cytoplasmic face of the nuclear pore complex could possibly facilitate virus-like particle docking, in a similar manner to their recruitment and binding of host karyopherins prior to nuclear import [84–87]. Multiple Ty3 proteins (Gag3, p27 and CA) interact directly with nucleoporins, and the integrase of Ty1 and Ty3 contain nuclear localization signals [25,41–44]. Therefore, it seems likely that Ty proteins interact directly with nucleoporins, making it plausible that evolutionary selection could be acting to alter these physical interactions.

The evolutionary relationship between yeast and Tys is complex. The intracellular lifecycle and ubiquity of Tys would suggest that Tys have been co-evolving with the *Saccharomyces* genus for many millions of years [1,2]. Ty copy number varies greatly between different strains and species of *Saccharomyces* yeasts and there is likely a dynamic cycle of Ty gain and loss. Indeed, certain families of Ty are completely absent from certain strains and species of *Saccharomyces* yeasts [1,65,88–90]. One example relevant to our findings is the apparent lack of Ty1 from *S*. *bayanus* [1], which is a complex hybrid species with genetic contributions from *S*. *cerevisiae*, *S*. *eubayanus*, and *S*. *uvarum* [91]. Our results show that *NUP84* from *S*. *bayanus* inhibits Ty1 mobility, this might have been protective against colonization by *S*. *cerevisiae* Ty1 during hybridization. The general persistence of Ty in *Saccharomyces* yeasts suggests that complete loss of Tys from a species is relatively rare, perhaps due to continued Ty introgression or transmission by sexual reproduction, which are potential mechanisms by which Tys can invade Ty-free or naive populations [1,90]. The error prone nature of the Ty reverse transcriptase and reverse transcription-mediated recombination can also generate Ty variants that could overcome host-encoded resistance mechanisms [92]. In contrast to the idea that Tys are completely parasitic, Ty mobility can drive the evolution of the yeast genome by changing gene regulation and expression by integrating in or near host genes. Tys can also facilitate gross chromosomal rearrangements of the host genome, including translocations and deletions, by way of homologous recombination between Ty integrated at different locations within host chromosomes [93–96]. Experimental systems have shown that Ty-mediated genome evolution can be observed in the laboratory [4], and would likely allow populations of *Saccharomyces* yeasts to rapidly respond to selective pressures found within the natural environment. Thus, in the context of the nuclear pore complex, there may be evolutionary selection to prevent Ty nuclear transit and excessive replication, but also selection against mutations that completely abrogate Ty mobility. The long-term association between *S*. *cerevisiae* and its cognate Tys would imply that this interaction has been optimized by evolutionary selection, perhaps to balance the damaging effects of excessive Ty mobility with the benefits of genome plasticity.

The nuclear pore is the gatekeeper of the nucleus, and it is antagonized by many pathogens and genetic parasites throughout eukaryotes. Recently, we have demonstrated that naturally occurring evolutionary differences between primate species in a nuclear pore component called RanBP2/Nup358 alter infection by simian immunodeficiency viruses (SIVs) [120]. We showed that differential interaction with RanBP2 in each host species drove SIV evolution as it transmitted between ape species, ultimately setting the stage for the zoonoses that yielded HIV-1. Remarkably, the very same nucleoporins that are under positive selection in yeast have also been shown to be essential to the replication of other retrotransposons and viruses. The fission yeast *Schizosaccharomyces pombe* ortholog of *NUP1* (*NUP124*) is required for mobility of the Ty3/gypsy-like element Tf1 and directly interacts with the Tf1-encoded Gag protein [97,98]. The human homologs of *NUP1* and *NUP116* (*NUP153* and *NUP98*, respectively) are important for viral replication in humans, including for HIV, HBV, HCV, and influenza virus [24,29,33,99–104]. Specifically, *NUP153* is an important determinant of HIV and HBV nuclear import, and its FG (phenylalanine-glycine)-repeat domain directly interacts with HIV capsid, via specific FG-repeats [24,33,105,106]. In *S*. *cerevisiae*, the FG-repeat region of Nup116p directly interacts *in vitro* with the Ty3-encoded protein Gag3, and truncation of *NUP116* decreases Ty3 mobility [25]. Collectively, this paints a picture of complex evolutionary pressures on nuclear pore genes across eukaryotes.

It appears that viral infections have broadly shaped the evolution of host genomes, affecting genes well beyond immunity loci [107]. The most classic example involves cellular entry receptors used by viruses to enter cells. These receptors are often under positive selection, resulting in highly species-specific interactions with viruses [108–112]. The nuclear pore complex is the gatekeeper of the nucleus just like cell surface receptors are gatekeepers of the cytoplasm. Our work in *Saccharomyces* yeasts provides a framework to further investigate the importance of the nuclear pore complex in modulating Ty mobility, and for a parallel investigation into the evolution of the orthologous nuclear pore complex of higher eukaryotes. It remains unknown how broadly viruses and genetic parasites are driving the evolution of important housekeeping proteins, but intriguing recent reports involving genes such as *XRN1* (involved in degradation of uncapped mRNAs; [11]), and DNA repair genes [113], suggest that this might be more common than previously appreciated.

## Materials and methods

### Plasmid construction

The *ACT1* intron (ACT1i) and an artificial intron (AI) [114] were amplified by PCR with included primer-encoded flanking homology to *GFP*. This PCR product was inserted directly after the ATG start codon at the 5’ end of *GFP* by the “yeast plasmid construction by homologous recombination” method (recombineering) [115]. *GFP*(AI) and *GFP*(ACT1i) were amplified by PCR and introduced into pAG423-GAL-*ccdB* using TOPO-TA and Gateway cloning strategies (Thermo Fisher) to create pPAR061 and pPAR063, respectively. *GFP*(ACT1i) was also placed under the control of the *CUP1* inducible promoter (456 bp upstream of *CUP1* were cloned directly from the genome of *S*. *cerevisiae*) using recombineering. pCUP1-*GFP*(ACT1i) was used to replace *HIS3*(AI) within pGTy1-HIS3(AI) to create pPAR078. pPAR101, pPAR104, pPAR145 and pPAR181 were constructed by using PCR to create DNA encoding FLAG-tagged Nup82p^DFY-LILLF^(1–458), Nup82p^DFY-LILLF^ and Nup82p (433–713) from pNOP-GFP-Nup82p^DFY-LILLF^ [77]. *MET17* was amplified directly from the genome of *S*. *cerevisiae*. All PCR fragments were subsequently cloned into pAG414-GPD-ccdB via pCR8 using TOPO-TA and Gateway cloning strategies (Thermo Fisher). To assay nuclear import using the strategy outlined by Marshall *et al*. we first subcloned the LexA-MBP-GAL4(AD) cassette from pJMB1076n [71] into the pAG413 plasmid backbone using recombineering, essentially changing the selective marker on the plasmid from *LEU2* to *HIS3*. For gene knockout, all plasmids were constructed by recombineering using *NUP82* and *NUP84* amplified from various *Saccharomyces* species. These nucleoporin genes were placed upstream of a selectable marker (*LEU2* or *KANMX6*) and the entire cassette flanked by 1000 bp of sequence encompassing the 5’ and 3’ untranslated regions of *NUP82* or *NUP84* from *S*. *cerevisiae*. pPAR240 was constructed by first amplifying a LexA operator sequence upstream of the β-galactosidase gene from *S*. *cerevisiae* L40. PCR products were designed to contain flanking homology to *ADE2* from *S*. *cerevisiae* and these PCR products were used to disrupt the *ADE2* gene within pRS422 to create pPAR240. The DNA sequences from all constructed plasmids can be found in S2 File. A list of all relevant plasmids can be found in S1 Table. A list of all relevant yeast strains can be found in S2 Table.

### Evolutionary analyses

Gene sequences from six species of *Saccharomyces* yeasts were obtained from publically available online resources. Maximum likelihood analysis of dN/dS was performed using the codeml program in PAML 4.1. Multiple protein sequence alignments were created and were manually curated to remove ambiguities before processing with PAL2NAL to produce accurate DNA alignments [116]. DNA alignments were fit to two models: M7 (codons fit to a beta distribution of dN/dS values, with dN/dS > 1 disallowed) and M8 (similar to model 7, but with dN/dS > 1 allowed). One model of codon frequency (f61) and a seed value of 0.4 for dN/dS (ω) was used (S1 File). Likelihood ratio tests were performed to evaluate which model of evolution the data fit significantly better with positive selection and inferred if we can reject M7 in favor of M8 with a p<0.05. REL and FEL codon based models were also used to detect sites under positive selection as implemented by the HyPhy package using the best substitution models chosen by Akaike information criterion (AIC) using the phylogenetic tree (Newick format):

((((*S*. *paradoxus*, *S*. *cerevisiae*), *S*. *mikatae*), *S*. *kudriavzevii*), *S*. *arboricolus*, *S*. *bayanus*) (S1 File).

### Strain construction

Standard methodologies for PCR-based gene knockout and replacement were used to create all *NUP84* strains in BY4741 (YPAR0130-0133) [117]. Strains YPAR0135-0138 were engineered to encode a LexA operator sequence upstream of the β-galactosidase gene, and were constructed by the disruption of the genomic copy of *ADE2* using a PCR cassette amplified from pPAR240. Clones selected for their ability to grow on a medium lacking uracil and inability to grown on a medium lacking adenine. *NUP82* gene replacement utilized a SceI-based method to increase the efficiency of the integration of *NUP82* and *KANMX6* by generating DNA double-stranded breaks at the *NUP82* locus in *S*. *cerevisiae* (personal communication, Dr. C.M. Yellman). Using a diploid heterozygous knockout strain of *NUP82* [78], *KANMX6* at the *NUP82* locus was replaced with the *URA3* gene from *K*. *lactis* flanked by SceI sites amplified by PCR from pCMY-IT3. Gene replacement was carried out by the concomitant expression of SceI from pGAL1-SCEH and the LiAc transformation of a PCR-derived cassette encoding *NUP82-KANMX6*. *NUP82/nup82*Δ::*NUP82-KANMX6* clones were selected by their ability to grow in the presence of 400 μg mL^-1^ G418 and their resistance to 5-FOA (0.1% w/v). Haploid clones were isolated from the engineered diploid strains using the SGA selection protocol as described previously [78]. The correct insertions were confirmed by PCR of genomic DNA of the *NUP82* locus to create strains YPAR0139, YPAR0143, YPAR0141 and YPAR0142. A PCR cassette was used to disrupt *HIS3* in YPAR0139, YPAR0143, YPAR0141 and YPAR0142, clones were selected for their ability to grow on a medium containing hygromycin and inability to grown on a medium lacking histidine to produce strains YPAR0143, YPAR0145, YPAR0147 and YPAR0149.

### Splicing of the ACT1 intron and insertion of an artificial intron within the GFP gene

Plasmids pPAR063 and pPAR061 were used to produce *GFP* transcripts containing either the *ACT1* intron (ACT1i) or an artificial intron (AI) [114], respectively, by induction from a galactose inducible promoter. Cultures were grown to mid-log phase in liquid culture with raffinose as the sole carbon source. At OD_600_ of ~1, galactose or dextrose was added to a final concentration of 2% and the cultures grown at 30°C for 2 h. Total RNA was extracted from these cultures (~2 x 10^7^ cells) using the RNeasy RNA extraction kit (Qiagen). 5 μg of RNA was treated with 1 U of DNase I at 37°C for 10 min before heat inactivation at 75°C for 10 min. RNA samples were then subject to two-step RT-PCR using Superscript III with the *GFP-*specific primers:

5’-AAGCTGACCCTGAAGTTCATCTGC-3’ and

5’-CGTTGTGGCTGTTGTAGTTGTACTCC-3’.

### Ty1 mobility assays

Yeast strains to be assayed for their ability to support Ty1 mobility were transformed with either pPAR078 (GFP flow cytometry method), pGTy1(*HIS3*(AI)) [73] or pBDG606 [51]. To detect Ty1 mobility, single colonies from *S*. *cerevisiae* transformed with each plasmid were isolated for each experiment. Each experiment was performed at least three times. Colonies were first grown for 24 h in 2 mL raffinose -uracil complete medium at 30°C with agitation. 1 x 10^5^ cells from the saturated cultures were used to inoculate 15 mL of -uracil complete medium with either 2% galactose or 2% raffinose with 0.02% galactose, followed by growth for 5 days at room temperature with agitation. For pGTy1(*HIS3*(AI)) or pBDG606: Cultures were serially diluted and plated onto a -uracil complete medium and a -uracil -histidine complete medium, both with dextrose as a carbon source. Colonies were counted after 2 days growth at 30°C and the percentage mobility was calculated. For pPAR078: Cultures were diluted and allowed to reach early log phase growth (OD_600_ ~0.05) before the addition of CuSO_4_ to a final concentration of 0.5 mM. Cultures were grown for 9 h at 30°C before assaying for the presence of live, GFP positive cells using a BD LSRII Fortessa flow cytometer (San Jose, CA) running FACSDiva software (v6.1.3). GFP excitation was observed with a blue, 488 nm laser, while GFP emission was collected using 530/30 nm band pass filter and 502 nm long pass filter. Propidium iodide (PI) excitation was observed with a yellow-green, 561 nm laser, while PI emission was collected using 660/20 nm band pass filter and a 635 nm long pass filter. 100,000 gated events were collected using a forward scatter vs side scatter dot plot, with forward scatter showing relative particle size and side scatter showing internal complexity. All subsequent plots were generated from this gated population. Live cells were gated by staining cell populations with PI (final concentration 0.1 μg mL^-1^) and GFP positive populations were gated by comparison with GFP negative populations of cells. Analysis of flow cytometry data was performed using FlowJo version 9.7.6.

### Ty3 mobility assay

For quantification of Ty3 mobility, yeast cells were transformed with pPS3858, a *URA3* marked galactose inducible Ty3-HIS3 [70,74–76]. The *HIS3* gene is located at the end of *POL* and is anti-sense to Ty3, except for an artificial intron which is sense. The sense intron prevents production of His3p until after the full-length Ty3 RNA is transcribed, spliced, reverse transcribed and integrated into the genome. Colony transformants were selected on a synthetic medium with 2% glucose (SD) complete with amino acids but lacking uracil. Single colonies were inoculated into 2 mL of synthetic raffinose (–uracil) and grown for 24 h. Cultures were then brought to 5 mL and grown for ~8 h, after which OD_600_ was measured and cultures were diluted back to an OD_600_ of ~0.02 in 4.5 mL and grown overnight. The following morning, 500 μL of 20% galactose (2% final) was added to induce Ty3 expression; after 8 h of induction cultures were pelleted, washed in SD medium, serially diluted, and plated on both SD plates lacking histidine for growth of transposed cells and also YPD plates to determine total live cell counts.

### Nup82p^DFY-LILLF^ expression assays

Plasmids constitutively expressing either Nup82p^DFY-LILLF^, Nup82p truncation mutants or Met17p were introduced into a strain containing a Ty1 mobility reporter plasmid. Mobility assays were carried out as previously described above, but with the use of a double-dropout complete medium to maintain both episomal vectors.

### Western blotting of Nup82p^DFY-LILLF^ and truncation mutants

Yeast lysates were prepared from 5 mL of stationary phase culture grown for 16 h in a yeast complete medium -leucine -tryptophan. Cell pellets were washed with 1 mL of chilled 25 mM Tris-HCl (pH 7.0), 10 mM sodium azide before incubation at 100°C for 3 min. 50 μL of SDS sample loading buffer (100 mM Tris-HCl, 5% SDS, 10% glycerol, 0.1% bromophenol blue, 2% β-mercaptoethanol, pH 6.8) was added to the boiled pellet with 200 μL of acid-washed glass beads (0.5 mm). Samples were vortexed for 10 min to disrupt yeast cells before the addition of another 80 μL of SDS sample loading buffer. Glass beads were pelleted by centrifugation (1500 × g, 2 min). 30 μL of each sample was loaded directly onto a precast Tris-glycine 10% SDS-PAGE gel (Biorad). Flag-tagged *NUP82* mutants were detected via Western blot using a 1:4000 dilution of a primary mouse monoclonal anti-flag (Syd Labs #M20008). Secondary detection was carried out using a 1:2000 dilution of a goat anti-mouse horseradish peroxidase conjugated antibody (Thermo #32430).

### Nuclear import assays

A LexA-MBP-GAL4(AD) fusion protein with or without an SV40 nuclear localization signal [71] was used to measure the efficiency of nuclear import within *S*. *cerevisiae* L40 or BY4741. 5 mL of a glucose-supplemented synthetic complete medium lacking the appropriate amino acid and grown overnight at 30°C with agitation. Cells were collected by centrifugation at 4000 × g for 30 seconds and the cell pellets suspended in 750 μL of ice-cold ddH_2_O. Washed cells were again collected by centrifugation (13,000 x g for 30 seconds), and soluble proteins extracted by Y-PER buffer as per manufacturer’s instructions (Thermo). The lysate was assayed for β-galactosidase activity as described previously [118].

### Fluorescence microscopy

The steady-state import of GFP-NLS was monitored within BY4741 transformed with pEB0836 as described previously [72].

### Detection of Ty1 genomic integrations by Southern blotting

The detection of the integration of Ty1 containing *GFP* by Southern blotting was performed as previously described [73], in the various *NUP84-*complemented or deletion strains of *S*. *cerevisiae*. Total DNA was extracted from cell cultures using phenol:chloroform and ethanol precipitation, after 5 days of induction, as described in the Ty1 mobility assay protocol above. Southern blotting was carried out after agarose gel electrophoresis, as described previously [119], using Hybond-XL membranes (GE healthcare).

### Promoter activity assay

To assay the activity of the *GAL1* promoter we expressed *GFP* under the control of the *GAL1* promoter and monitored the increase in the mean fluorescent intensity (MFI) compared to uninduced control cells. Cells were grown overnight to saturation at 30°C (CM -uracil, 2% raffinose) before being used to seed a 10 mL culture that was grown to log phase (OD_600_ 0.1–0.5). Each 10 mL log phase culture was divided into two 5 mL cultures, supplemented with either 2% galactose or dextrose (final concentration) and grown for 6 h. Cultures were assayed for GFP fluorescence by flow cytometry using the same instrumentation as described above. The activity of the *CUP1* promoter was assayed by analyzing MFI data derived from Ty1-GFP mobility assays.

## Supporting information

S1 Fig Nucleoporins and karyopherins are important for Ty mobility, but karyopherins are not evolving rapidly. (A) A summary of whole genome studies that have identified nucleoporins and karyopherins important for Ty1 and Ty3 mobility [51–57]. (B) Results from PAML analysis surveying karyopherins for signatures of positive selection, comparing a codon model of purifying selection (M7) to a codon model of positive selection (M8). No karyopherins had a p<0.05.
(TIF)

S2 Fig Ty1 mobility is generally reduced when assayed from a single copy reporter plasmid in *S. cerevisiae* expressing orthologous *NUP84*. (A) Relative mobility of Ty1 was assayed with a single copy plasmid within strains complemented with *NUP84* from different *Saccharomyces* species using the auxotrophic marker *HIS3*. Ty1 transcription was initiated by high (2%) or low (0.02%) concentrations of galactose (B) Averaged percentage of cells that scored positive for Ty1 mobility (Y axis) comparing low- (centromeric; CEN) and high- (2-micron plasmid; 2μm) copy number plasmids with the expression of Ty1 driven by high or low levels of expression via the *GAL1* promoter (error bars: standard error, n>4). For the GFP assay this was calculated as the overall percentage of GFP +ve cells in the total population. For the CEN plasmid, this was calculated as the percentage of cells that could grow on a complete medium lacking histidine.
(TIF)

S3 Fig The construction of *S. cerevisiae* strains expressing heterospecific *NUP82*. The *KANMX6* gene within a diploid strain of *S*. *cerevisiae* heterozygous for *KANMX6* at one *NUP82* locus (A) was replaced with the *URA3* gene from *K*. *lactis* flanked by SceI sites (B-C). SceI restriction endonuclease was used to create double-stranded DNA breaks at the *URA3*-containing *NUP82* locus, which was simultaneously repaired by a PCR-derived cassette encoding heterospecific *NUP82* and *KANMX6* (D-E). Haploid clones were isolated using the SGA selection protocol [78] (F-G).
(TIF)

S4 Fig The evolution of *NUP82* does not impact GFP production from different promoters. The effect of *NUP84* complementation on the ability of *S*. *cerevisiae* to express GFP from the promoters used in our Ty1 *GFP*-based reporter (*GAL1* or *CUP1* promoters).
(TIF)

S1 File Evolutionary analysis of genes involved nucleocytoplasmic transport. This spreadsheet summarizes the results from all of the evolutionary analyses that were performed.
(XLSX)

S2 File Plasmid sequences. This file contains sequences of plasmids constructed as part of this study.
(TXT)

S1 Table Plasmid sequences. A table containing the names and descriptions of all plasmids used in this study and their origins.
(DOCX)

S2 Table Yeast strains. A table containing the names and descriptions of all yeast strains used in this study and their origins.
(DOCX)

## References

[1] Liti G, Peruffo A, James SA, Roberts IN, Louis EJ. Inferences of evolutionary relationships from a population survey of LTR-retrotransposons and telomeric-associated sequences in the *Saccharomyces sensu stricto* complex. Yeast. 2005;22: 177–192. doi: 10.1002/yea.1200 15704235 10.1002/yea.1200

[2] Dujon B. Yeasts illustrate the molecular mechanisms of eukaryotic genome evolution. Trends Genet. 2006;22: 375–387. doi: 10.1016/j.tig.2006.05.007 16730849 10.1016/j.tig.2006.05.007

[3] Garfinkel DJ. Genome evolution mediated by Ty elements in *Saccharomyces*. Cytogenet Genome Res. 2005;110: 63–69. doi: 10.1159/000084939 16093659 10.1159/000084939

[4] Gresham D, Desai MM, Tucker CM, Jenq HT, Pai DA, Ward A, et al The repertoire and dynamics of evolutionary adaptations to controlled nutrient-limited environments in yeast. PLoS Genet. 2008;4: e1000303 doi: 10.1371/journal.pgen.1000303 19079573 10.1371/journal.pgen.1000303 PMC2586090

[5] Scheifele LZ, Cost GJ, Zupancic ML, Caputo EM, Boeke JD. Retrotransposon overdose and genome integrity. Proc Natl Acad Sci U S A. National Acad Sciences; 2009;106: 13927–13932. doi: 10.1073/pnas.0906552106 19666515 10.1073/pnas.0906552106 PMC2728997

[6] Wilke CM, Adams J. Fitness effects of Ty transposition in *Saccharomyces cerevisiae*. Genetics. 1992;131: 31–42. 1317316 10.1093/genetics/131.1.31 PMC1204961

[7] Nishida Y, Pachulska-Wieczorek K, Błaszczyk L, Saha A, Gumna J, Garfinkel DJ, et al Ty1 retrovirus-like element Gag contains overlapping restriction factor and nucleic acid chaperone functions. Nucleic Acids Res. 2015;43: 7414–7431. doi: 10.1093/nar/gkv695 26160887 10.1093/nar/gkv695 PMC4551931

[8] Matsuda E, Garfinkel DJ. Posttranslational interference of Ty1 retrotransposition by antisense RNAs. Proc Natl Acad Sci U S A. 2009;106: 15657–15662. doi: 10.1073/pnas.0908305106 19721006 10.1073/pnas.0908305106 PMC2735561

[9] Saha A, Mitchell JA, Nishida Y, Hildreth JE, Ariberre JA, Gilbert WV, et al A trans-Dominant Form of Gag Restricts Ty1 Retrotransposition and Mediates Copy Number Control. Sundquist WI, editor. J Virol. 2015;89: 3922–3938. doi: 10.1128/JVI.03060-14 25609815 10.1128/JVI.03060-14 PMC4403431

[10] Feng G, Leem YE, Levin HL. Transposon integration enhances expression of stress response genes. Nucleic Acids Res. 2013;41: 775–789. doi: 10.1093/nar/gks1185 23193295 10.1093/nar/gks1185 PMC3553992

[11] Rowley PA, Ho B, Bushong S, Johnson A, Sawyer SL. *XRN1* Is a Species-Specific Virus Restriction Factor in Yeasts. PLoS Pathog. 2016;12: e1005890 doi: 10.1371/journal.ppat.1005890 10.1371/journal.ppat.1005890 PMC5053509 27711183

[12] Sawyer SL, Malik HS. Positive selection of yeast nonhomologous end-joining genes and a retrotransposon conflict hypothesis. Proc Natl Acad Sci U S A. 2006;103: 17614–17619. doi: 10.1073/pnas.0605468103 17101967 10.1073/pnas.0605468103 PMC1693795

[13] Cohen S, Au S, Panté N. How viruses access the nucleus. Biochim Biophys Acta. 2011;1813: 1634–1645. doi: 10.1016/j.bbamcr.2010.12.009 21167871 10.1016/j.bbamcr.2010.12.009

[14] Whittaker G. Virus nuclear import. Adv Drug Deliv Rev. 2003;55: 733–747. doi: 10.1016/S0169-409X(03)00051-6 12788537 10.1016/s0169-409x(03)00051-6

[15] Mettenleiter TC. Breaching the Barrier—The Nuclear Envelope in Virus Infection. J Mol Biol. Elsevier Ltd; 2016;428: 1949–1961. doi: 10.1016/j.jmb.2015.10.001 26522933 10.1016/j.jmb.2015.10.001

[16] Neumann N, Lundin D, Poole AM. Comparative genomic evidence for a complete nuclear pore complex in the last eukaryotic common ancestor. PLoS ONE. 2010;5: e13241 doi: 10.1371/journal.pone.0013241 20949036 10.1371/journal.pone.0013241 PMC2951903

[17] Tamura K, Fukao Y, Iwamoto M, Haraguchi T, Hara-Nishimura I. Identification and characterization of nuclear pore complex components in *Arabidopsis thaliana*. Plant Cell. American Society of Plant Biologists; 2010;22: 4084–4097. doi: 10.1105/tpc.110.079947 21189294 10.1105/tpc.110.079947 PMC3027183

[18] DeGrasse JA, DuBois KN, Devos D, Siegel TN, Sali A, Field MC, et al Evidence for a shared nuclear pore complex architecture that is conserved from the last common eukaryotic ancestor. Mol Cell Proteomics. 2009;8: 2119–2130. doi: 10.1074/mcp.M900038-MCP200 19525551 10.1074/mcp.M900038-MCP200 PMC2742445

[19] Rout MP, Aitchison JD, Suprapto A, Hjertaas K, Zhao Y, Chait BT. The yeast nuclear pore complex: composition, architecture, and transport mechanism. J Cell Biol. 2000;148: 635–651. 10684247 10.1083/jcb.148.4.635 PMC2169373

[20] Aitchison JD, Rout MP. The Yeast Nuclear Pore Complex and Transport Through It. Genetics. 2012;190: 855–883. doi: 10.1534/genetics.111.127803 22419078 10.1534/genetics.111.127803 PMC3296253

[21] Lin DH, Stuwe T, Schilbach S, Rundlet EJ, Perriches T, Mobbs G, et al Architecture of the symmetric core of the nuclear pore. Science. 2016;352: aaf1015–aaf1015. doi: 10.1126/science.aaf1015 27081075 10.1126/science.aaf1015 PMC5207208

[22] Alber F, Dokudovskaya S, Veenhoff LM, Zhang W, Kipper J, Devos D, et al The molecular architecture of the nuclear pore complex. Nature. 2007;450: 695–701. doi: 10.1038/nature06405 18046406 10.1038/nature06405

[23] Wente SR, Rout MP. The nuclear pore complex and nuclear transport. Cold Spring Harb Perspect Biol. 2010;2: a000562 doi: 10.1101/cshperspect.a000562 20630994 10.1101/cshperspect.a000562 PMC2944363

[24] Matreyek KA, Yucel SS, Li X, Engelman A. Nucleoporin NUP153 Phenylalanine-Glycine Motifs Engage a Common Binding Pocket within the HIV-1 Capsid Protein to Mediate Lentiviral Infectivity. Luban J, editor. PLoS Pathog. 2013;9: e1003693 doi: 10.1371/journal.ppat.1003693 24130490 10.1371/journal.ppat.1003693 PMC3795039

[25] Beliakova-Bethell N, Terry LJ, Bilanchone V, DaSilva R, Nagashima K, Wente SR, et al Ty3 nuclear entry is initiated by viruslike particle docking on GLFG nucleoporins. J Virol. 2009;83: 11914–11925. doi: 10.1128/JVI.01192-09 19759143 10.1128/JVI.01192-09 PMC2772691

[26] Schaller T, Ocwieja KE, Rasaiyaah J, Price AJ, Brady TL, Roth SL, et al HIV-1 capsid-cyclophilin interactions determine nuclear import pathway, integration targeting and replication efficiency. Aiken C, editor. PLoS Pathog. 2011;7: e1002439 doi: 10.1371/journal.ppat.1002439 22174692 10.1371/journal.ppat.1002439 PMC3234246

[27] Bauer DW, Huffman JB, Homa FL, Evilevitch A. Herpes Virus Genome, The Pressure Is On. J Am Chem Soc. 2013;135: 11216–11221. doi: 10.1021/ja404008r 23829592 10.1021/ja404008r PMC4019375

[28] Strunze S, Engelke MF, Wang I-H, Puntener D, Boucke K, Schleich S, et al Kinesin-1-Mediated Capsid Disassembly and Disruption of the Nuclear Pore Complex Promote Virus Infection. Cell Host Microbe. Elsevier Inc; 2011;10: 210–223. doi: 10.1016/j.chom.2011.08.010 21925109 10.1016/j.chom.2011.08.010

[29] Schmitz A, Schwarz A, Foss M, Zhou L, Rabe B, Hoellenriegel J, et al Nucleoporin 153 Arrests the Nuclear Import of Hepatitis B Virus Capsids in the Nuclear Basket. Taylor J, editor. PLoS Pathog. 2010;6: e1000741–15. doi: 10.1371/journal.ppat.1000741 20126445 10.1371/journal.ppat.1000741 PMC2813275

[30] Fitzgerald KD, Semler BL. Re-localization of Cellular Protein SRp20 during Poliovirus Infection: Bridging a Viral IRES to the Host Cell Translation Apparatus. Racaniello V, editor. PLoS Pathog. 2011;7: e1002127–19. doi: 10.1371/journal.ppat.1002127 21779168 10.1371/journal.ppat.1002127 PMC3136463

[31] Matreyek K, Engelman A. Viral and Cellular Requirements for the Nuclear Entry of Retroviral Preintegration Nucleoprotein Complexes. Viruses. Multidisciplinary Digital Publishing Institute; 2013;5: 2483–2511. doi: 10.3390/v5102483 24103892 10.3390/v5102483 PMC3814599

[32] Bichel K, Price AJ, Schaller T, Towers GJ, Freund SMV, James LC. HIV-1 capsid undergoes coupled binding and isomerization by the nuclear pore protein NUP358. Retrovirology. 2013;10: 81 doi: 10.1186/1742-4690-10-81 23902822 10.1186/1742-4690-10-81 PMC3750474

[33] Di Nunzio F, Fricke T, Miccio A, Valle-Casuso JC, Perez P, Souque P, et al Nup153 and Nup98 bind the HIV-1 core and contribute to the early steps of HIV-1 replication. Virology. Elsevier; 2013;440: 8–18. doi: 10.1016/j.virol.2013.02.008 23523133 10.1016/j.virol.2013.02.008 PMC3860269

[34] Wickner RB, Fujimura T, Esteban R. Viruses and prions of *Saccharomyces cerevisiae*. Adv Virus Res. Elsevier; 2013;86: 1–36. doi: 10.1016/B978-0-12-394315-6.00001-5 23498901 10.1016/B978-0-12-394315-6.00001-5 PMC4141569

[35] Rowley PA. The frenemies within: viruses, retrotransposons and plasmids that naturally infect *Saccharomyces* yeasts. Yeast. 2017;49: 111 doi: 10.1002/yea.3234 10.1002/yea.3234 28387035

[36] King AMQ, Adams MJ, Lefkowitz EJ, Carstens EB. Virus Taxonomy Elsevier; 2011 doi: 10.1016/B978-0-12-384684-6.X0001-8

[37] Beauregard A, Curcio MJ, Belfort M. The take and give between retrotransposable elements and their hosts. Annu Rev Genet. 2008;42: 587–617. doi: 10.1146/annurev.genet.42.110807.091549 18680436 10.1146/annurev.genet.42.110807.091549 PMC2665727

[38] Patterson K, Sandmeyer S, Bilanchone V. Ty3, a Position-specific Retrotransposon in Budding Yeast. Microbiol Spectr. 2015;3: 1–29. doi: 10.1128/microbiolspec.MDNA3-0057-2014 26104707 10.1128/microbiolspec.MDNA3-0057-2014

[39] Curcio MJ, Lutz S, Lesage P. The Ty1 LTR-Retrotransposon of Budding Yeast, *Saccharomyces cerevisiae*. Microbiol Spectr. 2015;3: 1–35. doi: 10.1128/microbiolspec.MDNA3-0053-2014 25893143 10.1128/microbiolspec.MDNA3-0053-2014 PMC4399242

[40] Bilanchone V, Clemens K, Kaake R, Dawson AR, Matheos D, Nagashima K, et al Ty3 Retrotransposon Hijacks Mating Yeast RNA Processing Bodies to Infect New Genomes. Hopper A, editor. PLoS Genet. 2015;11: e1005528–29. doi: 10.1371/journal.pgen.1005528 26421679 10.1371/journal.pgen.1005528 PMC4589538

[41] Moore SP, Rinckel LA, Garfinkel DJ. A Ty1 integrase nuclear localization signal required for retrotransposition. Mol Cell Biol. 1998;18: 1105–1114. 9448008 10.1128/mcb.18.2.1105 PMC108823

[42] Kenna MA, Brachmann CB, Devine SE, Boeke JD. Invading the yeast nucleus: a nuclear localization signal at the C terminus of Ty1 integrase is required for transposition in vivo. Mol Cell Biol. 1998;18: 1115–1124. 9448009 10.1128/mcb.18.2.1115 PMC108824

[43] Lin SS, Nymark-McMahon MH, Yieh L, Sandmeyer SB. Integrase Mediates Nuclear Localization of Ty3. Mol Cell Biol. American Society for Microbiology; 2001;21: 7826–7838. doi: 10.1128/MCB.21.22.7826-7838.2001 11604517 10.1128/MCB.21.22.7826-7838.2001 PMC99952

[44] McLane LM, Pulliam KF, Devine SE, Corbett AH. The Ty1 integrase protein can exploit the classical nuclear protein import machinery for entry into the nucleus. Nucleic Acids Res. 2008;36: 4317–4326. doi: 10.1093/nar/gkn383 18586821 10.1093/nar/gkn383 PMC2490736

[45] Bridier-Nahmias A, Tchalikian-Cosson A, Baller JA, Menouni R, Fayol H, Flores A, et al Retrotransposons. An RNA polymerase III subunit determines sites of retrotransposon integration. Science. 2015;348: 585–588. doi: 10.1126/science.1259114 25931562 10.1126/science.1259114

[46] Kirchner J, Connolly CM, Sandmeyer SB. Requirement of RNA polymerase III transcription factors for in vitro position-specific integration of a retroviruslike element. Science. 1995;267: 1488–1491. 7878467 10.1126/science.7878467

[47] Checkley MA, Mitchell JA, Eizenstat LD, Lockett SJ, Garfinkel DJ. Ty1 Gag Enhances the Stability and Nuclear Export of Ty1 mRNA. Traffic. 2012;14: 57–69. doi: 10.1111/tra.12013 22998189 10.1111/tra.12013 PMC3548082

[48] Sironi M, Cagliani R, Forni D, Clerici M. Evolutionary insights into host–pathogen interactions from mammalian sequence data. Nat Rev Genet. Nature Publishing Group; 2015;16: 224–236. doi: 10.1038/nrg3905 25783448 10.1038/nrg3905 PMC7096838

[49] Duggal NK, Emerman M. Evolutionary conflicts between viruses and restriction factors shape immunity. Nat Rev Immunol. 2012;12: 687–695. doi: 10.1038/nri3295 22976433 10.1038/nri3295 PMC3690816

[50] Meyerson NR, Sawyer SL. Two-stepping through time: mammals and viruses. Trends Microbiol. 2011;19: 286–294. doi: 10.1016/j.tim.2011.03.006 21531564 10.1016/j.tim.2011.03.006 PMC3567447

[51] Dakshinamurthy A, Nyswaner KM, Farabaugh PJ, Garfinkel DJ. *BUD22* affects Ty1 retrotransposition and ribosome biogenesis in *Saccharomyces cerevisiae*. Genetics. 2010;185: 1193–1205. doi: 10.1534/genetics.110.119115 20498295 10.1534/genetics.110.119115 PMC2927749

[52] Griffith JL, Coleman LE, Raymond AS, Goodson SG, Pittard WS, Tsui C, et al Functional genomics reveals relationships between the retrovirus-like Ty1 element and its host *Saccharomyces cerevisiae*. Genetics. 2003;164: 867–879. 12871900 10.1093/genetics/164.3.867 PMC1462630

[53] Scholes DT, Banerjee M, Bowen B, Curcio MJ. Multiple regulators of Ty1 transposition in *Saccharomyces cerevisiae* have conserved roles in genome maintenance. Genetics. Genetics Society of America; 2001;159: 1449–1465. 11779788 10.1093/genetics/159.4.1449 PMC1461915

[54] Nyswaner KM, Checkley MA, Yi M, Stephens RM, Garfinkel DJ. Chromatin-associated genes protect the yeast genome from Ty1 insertional mutagenesis. Genetics. 2008;178: 197–214. doi: 10.1534/genetics.107.082602 18202368 10.1534/genetics.107.082602 PMC2206071

[55] Risler JK, Kenny AE, Palumbo RJ, Gamache ER, Curcio MJ. Host co-factors of the retrovirus-like transposon Ty1. Mobile DNA. BioMed Central; 2012;3: 12 doi: 10.1186/1759-8753-3-12 22856544 10.1186/1759-8753-3-12 PMC3522557

[56] Irwin B, Aye M, Baldi P, Beliakova-Bethell N, Cheng H, Dou Y, et al Retroviruses and yeast retrotransposons use overlapping sets of host genes. Genome Res. 2005;15: 641–654. doi: 10.1101/gr.3739005 15837808 10.1101/gr.3739005 PMC1088292

[57] Aye M, Irwin B, Beliakova-Bethell N, Chen E, Garrus J, Sandmeyer S. Host factors that affect Ty3 retrotransposition in *Saccharomyces cerevisiae*. Genetics. 2004;168: 1159–1176. doi: 10.1534/genetics.104.028126 15579677 10.1534/genetics.104.028126 PMC1448793

[58] Maxwell PH, Curcio MJ. Host Factors That Control Long Terminal Repeat Retrotransposons in *Saccharomyces cerevisiae*: Implications for Regulation of Mammalian Retroviruses. Eukaryotic Cell. 2007;6: 1069–1080. doi: 10.1128/EC.00092-07 17496126 10.1128/EC.00092-07 PMC1951103

[59] Fischer J, Teimer R, Amlacher S, Kunze R, Hurt E. Linker Nups connect the nuclear pore complex inner ring with the outer ring and transport channel. Nat Struct Mol Biol. 2015;22: 774–781. doi: 10.1038/nsmb.3084 26344569 10.1038/nsmb.3084

[60] Stuwe T, Bley CJ, Thierbach K, Petrovic S, Schilbach S, Mayo DJ, et al Architecture of the fungal nuclear pore inner ring complex. Science. 2015;350: 56–64. doi: 10.1126/science.aac9176 26316600 10.1126/science.aac9176 PMC4826903

[61] Hurst LD. The Ka/Ks ratio: diagnosing the form of sequence evolution. Trends Genet. 2002;18: 486 12175810 10.1016/s0168-9525(02)02722-1

[62] Yang Z. Adaptive molecular evolution Balding DJ, Bishop M, Cannings C, editors. Handbook of statistical genetics. Wiley Online Library; 2001;: 327–350.

[63] Yang ZH, Nielsen R, Goldman N, Pedersen A. Codon-substitution models for heterogeneous selection pressure at amino acid sites. Genetics. Genetics Society of America; 2000;155: 431–449. 10790415 10.1093/genetics/155.1.431 PMC1461088

[64] Yang Z. PAML 4: Phylogenetic analysis by maximum likelihood. Mol Biol Evol. 2007;24: 1586–1591. doi: 10.1093/molbev/msm088 17483113 10.1093/molbev/msm088

[65] Liti G, Carter DM, Moses AM, Warringer J, Parts L, James SA, et al Population genomics of domestic and wild yeasts. Nature. 2009;458: 337–341. doi: 10.1038/nature07743 19212322 10.1038/nature07743 PMC2659681

[66] Naumov G, Naumova E, Masneuf-Pomarède I. Genetic identification of new biological species *Saccharomyces arboricolus* Wang et Bai. Antonie Van Leeuwenhoek. 2010;98: 1–7. doi: 10.1007/s10482-010-9441-5 20379848 10.1007/s10482-010-9441-5

[67] Scannell DR, Zill OA, Rokas A, Payen C, Dunham MJ, Eisen MB, et al The awesome power of yeast evolutionary genetics: New genome sequences and strain resources for the *Saccharomyces sensu stricto* genus. G3 (Bethesda). 2011;1: 11–25. doi: 10.1534/g3.111.000273 22384314 10.1534/g3.111.000273 PMC3276118

[68] Pond SLK, Frost SDW. Datamonkey: rapid detection of selective pressure on individual sites of codon alignments Bioinformatics Oxford University Press; 2005;21: 2531–2533. doi: 10.1093/bioinformatics/bti320 15713735 10.1093/bioinformatics/bti320

[69] Kosakovsky Pond SL, Frost SDW. Not so different after all: a comparison of methods for detecting amino acid sites under selection. Mol Biol Evol. Oxford University Press; 2005;22: 1208–1222. doi: 10.1093/molbev/msi105 15703242 10.1093/molbev/msi105

[70] Curcio MJ, Garfinkel DJ. Single-step selection for Ty1 element retrotransposition. Proc Natl Acad Sci U S A. 1991;88: 936–940. 1846969 10.1073/pnas.88.3.936 PMC50929

[71] Marshall KS, Zhang Z, Curran J, Derbyshire S, Mymryk JS. An improved genetic system for detection and analysis of protein nuclear import signals. BMC Mol Biol. 2007;8: 6 doi: 10.1186/1471-2199-8-6 17254328 10.1186/1471-2199-8-6 PMC1796550

[72] Kaffman A, Rank NM, O'Shea EK. Phosphorylation regulates association of the transcription factor Pho4 with its import receptor Pse1/Kap121. Genes Dev. 1998;12: 2673–2683. 9732266 10.1101/gad.12.17.2673 PMC317126

[73] Checkley MA, Nagashima K, Lockett SJ, Nyswaner KM, Garfinkel DJ. P-body components are required for Ty1 retrotransposition during assembly of retrotransposition-competent virus-like particles. Mol Cell Biol. 2010;30: 382–398. doi: 10.1128/MCB.00251-09 19901074 10.1128/MCB.00251-09 PMC2798465

[74] Chalker DL, Sandmeyer SB. Transfer RNA genes are genomic targets for de Novo transposition of the yeast retrotransposon Ty3. Genetics. Genetics Society of America; 1990;126: 837–850. 1963869 10.1093/genetics/126.4.837 PMC1204282

[75] Sadeghi N, Rütz ML, Menees TM. Thermal blockage of viruslike particle formation for the yeast retrotransposon Ty3 reveals differences in the cellular stress response. Arch Virol. 2001;146: 1919–1934. 11722014 10.1007/s007050170042

[76] Bilanchone VW, Claypool JA, Kinsey PT, Sandmeyer SB. Positive and negative regulatory elements control expression of the yeast retrotransposon Ty3. Genetics. Genetics; 1993;134: 685–700. 8394262 10.1093/genetics/134.3.685 PMC1205508

[77] Yoshida K, Seo H-S, Debler EW, Blobel G, Hoelz A. Structural and functional analysis of an essential nucleoporin heterotrimer on the cytoplasmic face of the nuclear pore complex. Proc Natl Acad Sci U S A. 2011;108: 16571–16576. doi: 10.1073/pnas.1112846108 21930948 10.1073/pnas.1112846108 PMC3189060

[78] Tong AHY. Systematic Genetic Analysis with Ordered Arrays of Yeast Deletion Mutants. Science. 2001;294: 2364–2368. doi: 10.1126/science.1065810 11743205 10.1126/science.1065810

[79] Xie X, Qiu W-G, Lipke PN. Accelerated and adaptive evolution of yeast sexual adhesins. Mol Biol Evol. 2011;28: 3127–3137. doi: 10.1093/molbev/msr145 21633112 10.1093/molbev/msr145 PMC3247809

[80] Greig D, Travisano M. The Prisoner's Dilemma and polymorphism in yeast *SUC* genes. Proc Biol Sci. 2004;271: S25–6. doi: 10.1098/rsbl.2003.0083 15101409 10.1098/rsbl.2003.0083 PMC1810003

[81] Smith C, Greig D. The cost of sexual signaling in yeast. Evolution. 2010;64: 3114–3122. doi: 10.1111/j.1558-5646.2010.01069.x 20584074 10.1111/j.1558-5646.2010.01069.x

[82] Bensasson D, Zarowiecki M, Burt A, Koufopanou V. Rapid evolution of yeast centromeres in the absence of drive. Genetics. 2008;178: 2161–2167. doi: 10.1534/genetics.107.083980 18430941 10.1534/genetics.107.083980 PMC2323805

[83] Pieczynska MD, Wloch-Salamon D, Korona R, de Visser JAGM. Rapid multiple-level coevolution in experimental populations of yeast killer and nonkiller strains. Evolution. 2016;70: 1342–1353. doi: 10.1111/evo.12945 27168531 10.1111/evo.12945

[84] Damelin M, Silver PA. Mapping interactions between nuclear transport factors in living cells reveals pathways through the nuclear pore complex. Mol Cell. 2000;5: 133–140. 10678175 10.1016/s1097-2765(00)80409-8

[85] Lutzmann M, Kunze R, Stangl K, Stelter P, Tóth KF, Böttcher B, et al Reconstitution of Nup157 and Nup145N into the Nup84 complex. J Biol Chem. 2005;280: 18442–18451. doi: 10.1074/jbc.M412787200 15741174 10.1074/jbc.M412787200

[86] Patel SS, Rexach MF. Discovering Novel Interactions at the Nuclear Pore Complex Using Bead Halo: A Rapid Method for Detecting Molecular Interactions of High and Low Affinity at Equilibrium. Mol Cell Proteomics. 2007;7: 121–131. doi: 10.1074/mcp.M700407-MCP200 17897934 10.1074/mcp.M700407-MCP200

[87] Belanger KD, Gupta A, MacDonald KM, Ott CM, Hodge CA, Cole CM, et al Nuclear pore complex function in *Saccharomyces cerevisiae* is influenced by glycosylation of the transmembrane nucleoporin Pom152p. Genetics. 2005;171: 935–947. doi: 10.1534/genetics.104.036319 16118201 10.1534/genetics.104.036319 PMC1456851

[88] Moore SP, Liti G, Stefanisko KM, Nyswaner KM, Chang C, Louis EJ, et al Analysis of a Ty1-less variant of *Saccharomyces paradoxus*: the gain and loss of Ty1 elements. Yeast. 2004;21: 649–660. doi: 10.1002/yea.1129 15197730 10.1002/yea.1129

[89] Schacherer J, Shapiro JA, Ruderfer DM, Kruglyak L. Comprehensive polymorphism survey elucidates population structure of *Saccharomyces cerevisiae*. Nature. 2009;458: 342–345. doi: 10.1038/nature07670 19212320 10.1038/nature07670 PMC2782482

[90] Bleykasten-Grosshans C, Friedrich A, Schacherer J. Genome-wide analysis of intraspecific transposon diversity in yeast. BMC Genomics. BMC Genomics; 2013;14: 1–1. doi: 10.1186/1471-2164-14-1 23768249 10.1186/1471-2164-14-399 PMC4022208

[91] Libkind D, Hittinger CT, Valério E, Gonçalves C, Dover J, Johnston M, et al Microbe domestication and the identification of the wild genetic stock of lager-brewing yeast. Proc Natl Acad Sci U S A. 2011;108: 14539–14544. doi: 10.1073/pnas.1105430108 21873232 10.1073/pnas.1105430108 PMC3167505

[92] Bleykasten-Grosshans C, Jung PP, Fritsch ES, Potier S, de Montigny J, Souciet J-L. The Ty1 LTR-retrotransposon population in *Saccharomyces cerevisiae* genome: dynamics and sequence variations during mobility. FEMS Yeast Res. 2011;11: 334–344. doi: 10.1111/j.1567-1364.2011.00721.x 21272231 10.1111/j.1567-1364.2011.00721.x

[93] Zhang H, Zeidler AFB, Song W, Puccia CM, Malc E, Greenwell PW, et al Gene copy-number variation in haploid and diploid strains of the yeast *Saccharomyces cerevisiae*. Genetics. Genetics; 2013;193: 785–801. doi: 10.1534/genetics.112.146522 23307895 10.1534/genetics.112.146522 PMC3583998

[94] Roeder GS, Fink GR. DNA rearrangements associated with a transposable element in yeast. Cell. 1980;21: 239–249. 6250713 10.1016/0092-8674(80)90131-2

[95] Servant G, Pennetier C, Lesage P. Remodeling yeast gene transcription by activating the Ty1 long terminal repeat retrotransposon under severe adenine deficiency. Mol Cell Biol. 2008;28: 5543–5554. doi: 10.1128/MCB.00416-08 18591253 10.1128/MCB.00416-08 PMC2519716

[96] Chang S-L, Lai H-Y, Tung S-Y, Leu J-Y. Dynamic Large-Scale Chromosomal Rearrangements Fuel Rapid Adaptation in Yeast Populations. Fay JC, editor. PLoS Genet. 2013;9: e1003232–15. doi: 10.1371/journal.pgen.1003232 23358723 10.1371/journal.pgen.1003232 PMC3554576

[97] Balasundaram D, Benedik MJ, Morphew M, Dang VD, Levin HL. Nup124p is a nuclear pore factor of *Schizosaccharomyces pombe* that is important for nuclear import and activity of retrotransposon Tf1. Mol Cell Biol. 1999;19: 5768–5784. 10409764 10.1128/mcb.19.8.5768 PMC84427

[98] Dang VD, Levin HL. Nuclear import of the retrotransposon Tf1 is governed by a nuclear localization signal that possesses a unique requirement for the FXFG nuclear pore factor Nup124p. Mol Cell Biol. 2000;20: 7798–7812. 11003674 10.1128/mcb.20.20.7798-7812.2000 PMC86372

[99] Price AJ, Jacques DA, McEwan WA, Fletcher AJ, Essig S, Chin JW, et al Host Cofactors and Pharmacologic Ligands Share an Essential Interface in HIV-1 Capsid That Is Lost upon Disassembly. Cullen BR, editor. PLoS Pathog. 2014;10: e1004459–17. doi: 10.1371/journal.ppat.1004459 25356722 10.1371/journal.ppat.1004459 PMC4214760

[100] Brass AL, Dykxhoorn DM, Benita Y, Yan N, Engelman A, Xavier RJ, et al Identification of Host Proteins Required for HIV Infection Through a Functional Genomic Screen. Science. 2008;319: 921–926. doi: 10.1126/science.1152725 18187620 10.1126/science.1152725

[101] König R, Zhou Y, Elleder D, Diamond TL, Bonamy GMC, Irelan JT, et al Global Analysis of Host-Pathogen Interactions that Regulate Early-Stage HIV-1 Replication. Cell. 2008;135: 49–60. doi: 10.1016/j.cell.2008.07.032 18854154 10.1016/j.cell.2008.07.032 PMC2628946

[102] König R, Stertz S, Zhou Y, Inoue A, Hoffmann HH, Bhattacharyya S, et al Human host factors required for influenza virus replication. Nature. Nature Publishing Group; 2010;463: 813–817. doi: 10.1038/nature08699 20027183 10.1038/nature08699 PMC2862546

[103] Di Nunzio F, Danckaert A, Fricke T, Perez P, Fernandez J, Perret E, et al Human Nucleoporins Promote HIV-1 Docking at the Nuclear Pore, Nuclear Import and Integration. Chauhan A, editor. PLoS ONE. 2012;7: e46037–15. doi: 10.1371/journal.pone.0046037 23049930 10.1371/journal.pone.0046037 PMC3457934

[104] Lussignol M, Kopp M, Molloy K, Vizcay-Barrena G, Fleck RA, Dorner M, et al Proteomics of HCV virions reveals an essential role for the nucleoporin Nup98 in virus morphogenesis. Proc Natl Acad Sci U S A. National Acad Sciences; 2016;113: 2484–2489. doi: 10.1073/pnas.1518934113 26884193 10.1073/pnas.1518934113 PMC4780614

[105] Price AJ, Fletcher AJ, Schaller T, Elliott T, Lee K, KewalRamani VN, et al CPSF6 defines a conserved capsid interface that modulates HIV-1 replication. PLoS Pathog. 2012;8: e1002896 doi: 10.1371/journal.ppat.1002896 22956906 10.1371/journal.ppat.1002896 PMC3431306

[106] Matreyek KA, Engelman A. The Requirement for Nucleoporin NUP153 during Human Immunodeficiency Virus Type 1 Infection Is Determined by the Viral Capsid. J Virol. 2011;85: 7818–7827. doi: 10.1128/JVI.00325-11 21593146 10.1128/JVI.00325-11 PMC3147902

[107] Enard D, Cai L, Gwennap C, Petrov DA. Viruses are a dominant driver of protein adaptation in mammals. eLife. eLife Sciences Publications Limited; 2016;5 doi: 10.7554/eLife.12469 27187613 10.7554/eLife.12469 PMC4869911

[108] Ng M, Ndungo E, Kaczmarek ME, Herbert AS, Binger T, Kuehne AI, et al Filovirus receptor NPC1 contributes to species-specific patterns of ebolavirus susceptibility in bats. eLife. 2015;4: RRN1212 doi: 10.7554/eLife.11785 26698106 10.7554/eLife.11785 PMC4709267

[109] Kaelber JT, Demogines A, Harbison CE, Allison AB, Goodman LB, Ortega AN, et al Evolutionary reconstructions of the transferrin receptor of Caniforms supports canine parvovirus being a re-emerged and not a novel pathogen in dogs. Villarreal L, editor. PLoS Pathog. 2012;8: e1002666 doi: 10.1371/journal.ppat.1002666 22570610 10.1371/journal.ppat.1002666 PMC3342950

[110] Demogines A, Abraham J, Choe H, Farzan M, Sawyer SL. Dual Host-Virus Arms Races Shape an Essential Housekeeping Protein. Sugden B, editor. PLoS Biol. 2013;11: e1001571–13. doi: 10.1371/journal.pbio.1001571 23723737 10.1371/journal.pbio.1001571 PMC3665890

[111] Zhang ZD, Weinstock G, Gerstein M. Rapid Evolution by Positive Darwinian Selection in T-Cell Antigen CD4 in Primates. J Mol Evol. 2008;66: 446–456. doi: 10.1007/s00239-008-9097-1 18414925 10.1007/s00239-008-9097-1

[112] Martin C, Buckler-White A, Wollenberg K, Kozak CA. The avian XPR1 gammaretrovirus receptor is under positive selection and is disabled in bird species in contact with virus-infected wild mice. J Virol. American Society for Microbiology; 2013;87: 10094–10104. doi: 10.1128/JVI.01327-13 23843647 10.1128/JVI.01327-13 PMC3754004

[113] Lou DI, Kim ET, Meyerson NR, Pancholi NJ, Mohni KN, Enard D, et al An Intrinsically Disordered Region of the DNA Repair Protein Nbs1 Is a Species-Specific Barrier to Herpes Simplex Virus 1 in Primates. Cell Host Microbe. Elsevier Inc; 2016;20: 178–188. doi: 10.1016/j.chom.2016.07.003 27512903 10.1016/j.chom.2016.07.003 PMC4982468

[114] Yoshimatsu T, Nagawa F. Control of gene expression by artificial introns in *Saccharomyces cerevisiae*. Science. 1989;244: 1346–1348. 2544026 10.1126/science.2544026

[115] Schatz P. Plasmid construction by homologous recombination in yeast. Gene. 1987;58: 201–216. 2828185 10.1016/0378-1119(87)90376-3

[116] Suyama M, Torrents D, Bork P. PAL2NAL: robust conversion of protein sequence alignments into the corresponding codon alignments. Nucleic Acids Res. Oxford University Press; 2006;34: 609–612. doi: 10.1093/nar/gkl315 16845082 10.1093/nar/gkl315 PMC1538804

[117] Baudin A, Ozier-Kalogeropoulos O, Denouel A, Lacroute F, Cullin C. A simple and efficient method for direct gene deletion in *Saccharomyces cerevisiae*. Nucleic Acids Res. 1993;21: 3329–3330. 8341614 10.1093/nar/21.14.3329 PMC309783

[118] Kaiser C, Michaelis S, Mitchell A, Laboratory CSH. Methods in yeast genetics Cold Spring Harbor Laboratory Pr; 1994.

[119] Wong MCVL, Scott-Drew SRS, Hayes MJ, Howard PJ, Murray JAH. RSC2, Encoding a Component of the RSC Nucleosome Remodeling Complex, Is Essential for 2 m Plasmid Maintenance in *Saccharomyces cerevisiae*. Mol Cell Biol. 2002;22: 4218–4229. doi: 10.1128/MCB.22.12.4218-4229.2002 12024034 10.1128/MCB.22.12.4218-4229.2002 PMC133863

[120] Meyerson NR, Warren CJ, Vieira DASA, Diaz-Griffero F, Sawyer SL. Species-specific vulnerability of RanBP2 shaped the evolution of SIV as it transmitted in African apes. PLoS Pathogens. 2018;14 (3): e1006906 doi: 10.1371/journal.ppat.1006906 29518153 10.1371/journal.ppat.1006906 PMC5843284

