# Supplementary figures and images for: Control of yeast retrotransposons mediated through nucleoporin evolution
Source: PLoS Genet. 2018 Apr 25;14(4):e1007325. doi: 10.1371/journal.pgen.1007325 (PMC5918913; doi:10.1371/journal.pgen.1007325)

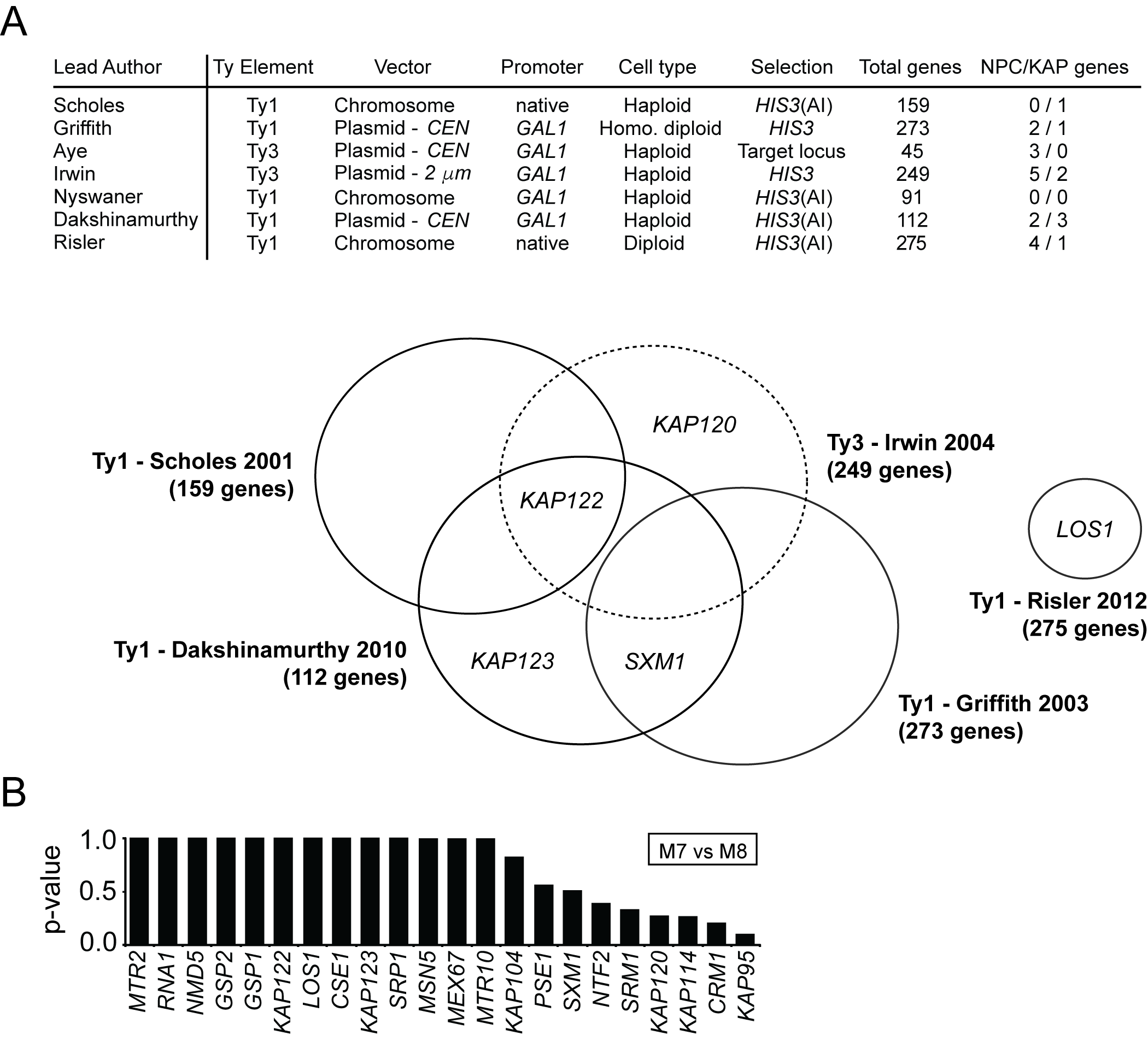

Supplement: S1 Fig — (A) A summary of whole genome studies that have identified nucleoporins and karyopherins important for Ty1 and Ty3 mobility [51–57]. (B) Results from PAML analysis surveying karyopherins for signatures of positive selection, comparing a codon model of purifying selection (M7) to a codon model of positive selection (M8). No karyopherins had a p<0.05. (TIF) [file pgen.1007325.s001.tif]

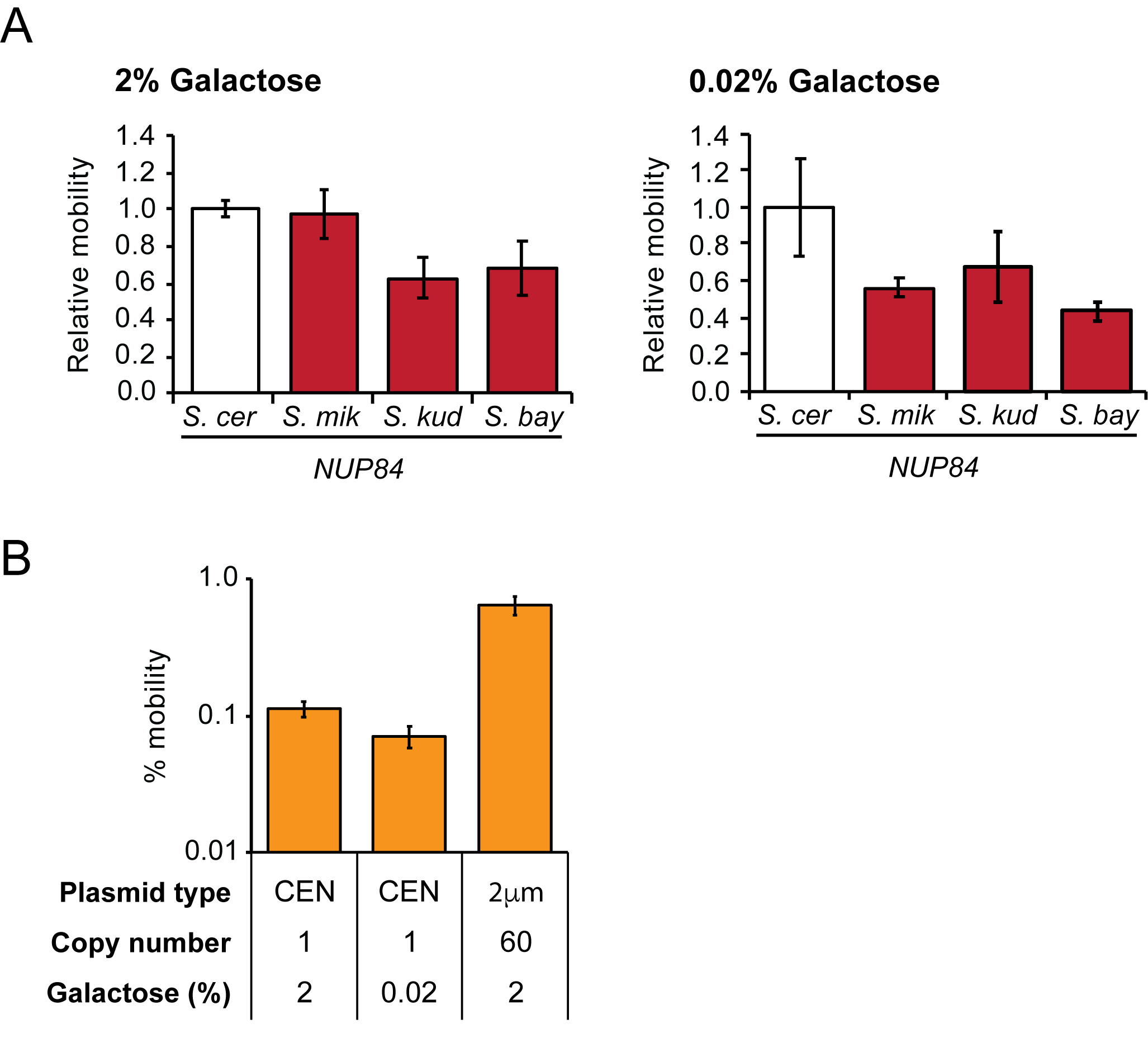

Supplement: S2 Fig — (A) Relative mobility of Ty1 was assayed with a single copy plasmid within strains complemented with NUP84 from different Saccharomyces species using the auxotrophic marker HIS3. Ty1 transcription was initiated by high (2%) or low (0.02%) concentrations of galactose (B) Averaged percentage of cells that scored positive for Ty1 mobility (Y axis) comparing low- (centromeric; CEN) and high- (2-micron plasmid; 2μm) copy number plasmids with the expression of Ty1 driven by high or low levels of expression via the GAL1 promoter (error bars: standard error, n>4). For the GFP assay this was calculated as the overall percentage of GFP +ve cells in the total population. For the CEN plasmid, this was calculated as the percentage of cells that could grow on a complete medium lacking histidine. (TIF) [file pgen.1007325.s002.tif]

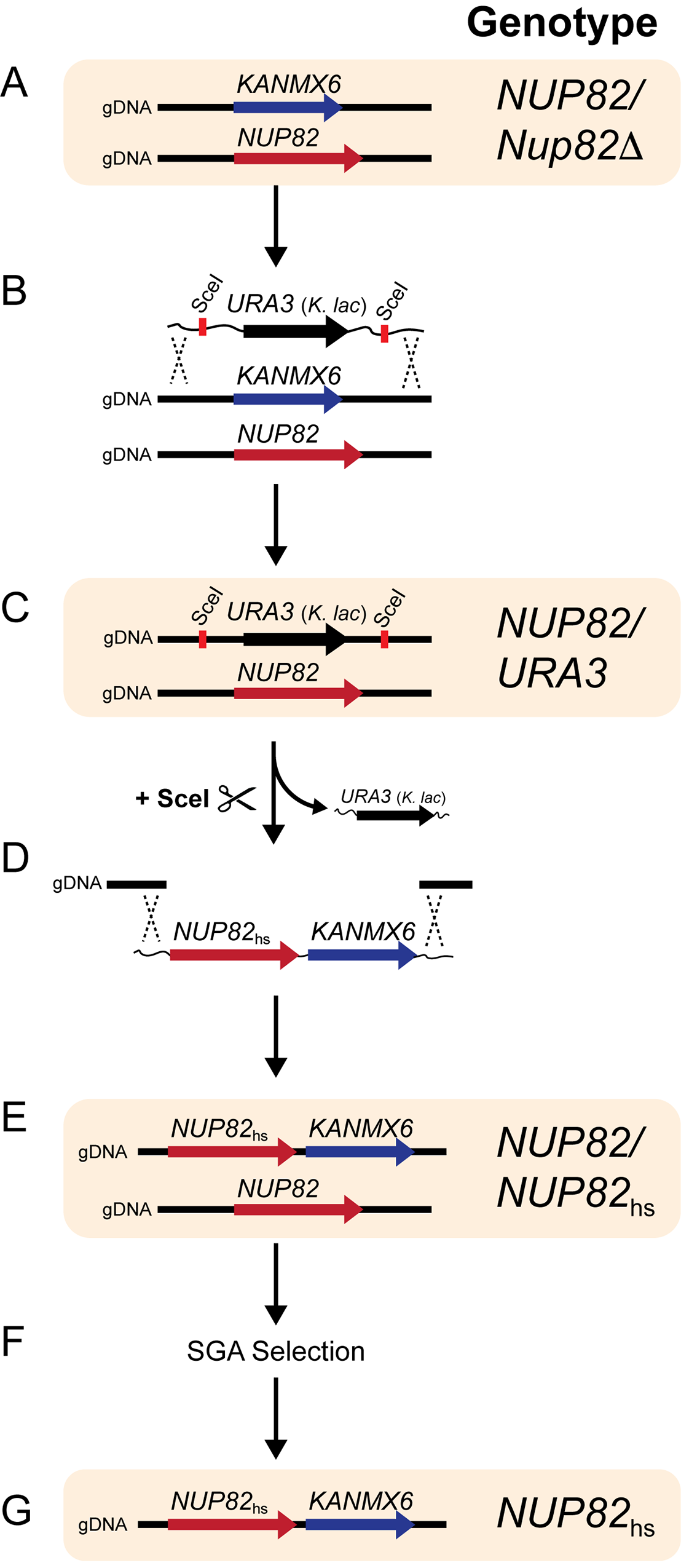

Supplement: S3 Fig — The KANMX6 gene within a diploid strain of S. cerevisiae heterozygous for KANMX6 at one NUP82 locus (A) was replaced with the URA3 gene from K. lactis flanked by SceI sites (B-C). SceI restriction endonuclease was used to create double-stranded DNA breaks at the URA3-containing NUP82 locus, which was simultaneously repaired by a PCR-derived cassette encoding heterospecific NUP82 and KANMX6 (D-E). Haploid clones were isolated using the SGA selection protocol [78] (F-G). (TIF) [file pgen.1007325.s003.tif]

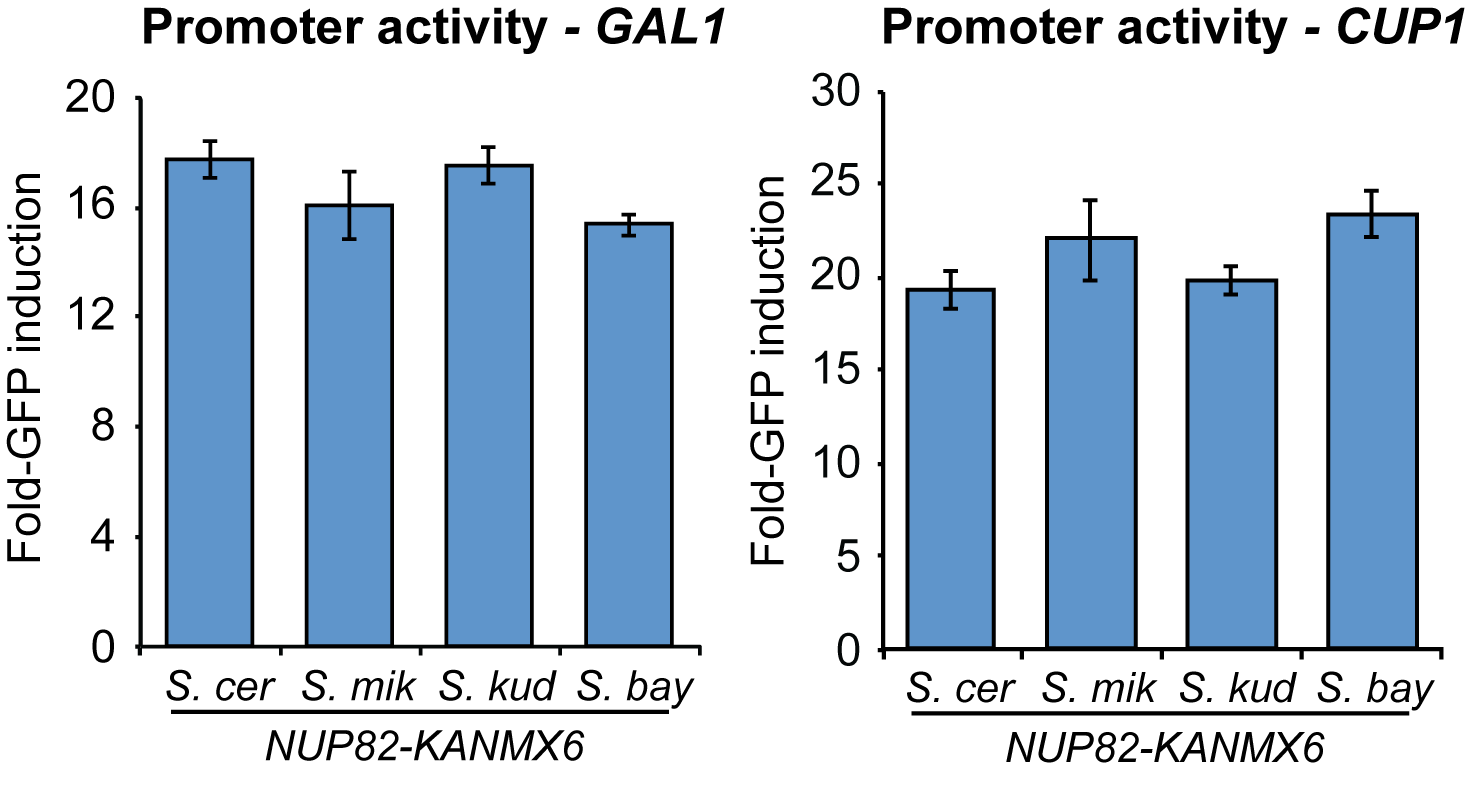

Supplement: S4 Fig — The effect of NUP84 complementation on the ability of S. cerevisiae to express GFP from the promoters used in our Ty1 GFP-based reporter (GAL1 or CUP1 promoters). (TIF) [file pgen.1007325.s004.tif]
