# Supplementary material for: Control of yeast retrotransposons mediated through nucleoporin evolution
Source: PLoS Genet. 2018 Apr 25;14(4):e1007325. doi: 10.1371/journal.pgen.1007325 (PMC5918913; doi:10.1371/journal.pgen.1007325)
Supplement: S1 Table — A table containing the names and descriptions of all plasmids used in this study and their origins. (DOCX) [file pgen.1007325.s007.docx]

**Table S1 – Plasmids**

| **Plasmid** | **Details** | **Markers** | **Source** |
| --- | --- | --- | --- |
| pNOP-GFP-*NUP82*^DFY-LILLF^ | *NUP82* mutant expression vector tagged with GFP. | *LEU2*, *CEN* | Yoshida et al. [1] |
| pJMB1076n | LexA-MBP-Gal4(AD) nuclear import assay +ve control. | *LEU2*, *CEN* | Marshall et al. [2] |
| pGTy1-HIS3(AI) | *GAL-1* inducible Ty1(*HIS3*(AI)) | *URA3, 2μm* | Curcio et al.[3] |
| pBDG606 | *GAL-1* inducible Ty3(*HIS3*(AI)) | *URA3, CEN* | Dakshinamurthy et al. [4] |
| pEB0836 | Pho4(140-156aa) fused to three GFP under the *PHO4* promoter | *URA3*, *CEN* | Kaffman et al. [5] |
| pPAR061 | GFP(AI) cloned into pAG423-GAL-ccdB | *HIS3, 2μm* | This study |
| pPAR063 | GFP(ACT1i) cloned into pAG423-GAL-ccdB | *HIS3, 2μm* | This study |
| pPAR078 | pGTy1-HIS3(AI) with HIS3(AI) replaced with CUP1-GFP(ACT1i) | *URA3, 2μm* | This study |
| pPAR101 | FLAG-*NUP82*^DFY-LILLF^(1-458) cloned into pAG414-GPD-ccdB | *TRP, CEN* | This study |
| pPAR104 | FLAG-*NUP82*^DFY-LILLF^ cloned into pAG414-GPD-ccdB | *TRP, CEN* | This study |
| pPAR145 | FLAG-*NUP82*(433-713) cloned into pAG414-GPD-ccdB | *TRP, CEN* | This study |
| pPAR181 | FLAG-*MET17* cloned into pAG414-GPD-ccdB | *TRP, CEN* | This study |
| pPAR198 | LexA-MBP-GAL4(AD) +SV40 NLS cloned into pAG413-GAL-eGFP-ccdB | *HIS3, CEN* | This study |
| pPAR199 | LexA-MBP-GAL4(AD) cloned into pAG413-GAL-eGFP-ccdB | *HIS3, CEN* | This study |
| pPAR200 | *NUP84* *S. cerevisiae* with *LEU2* and flanking sequence from the *NUP84 locus* | *URA3, LEU2*, *2μm* | This study |
| pPAR201 | *NUP82* *S. cerevisiae* with *KANMX6* and flanking sequence from the *NUP82 locus* | *URA3, LEU2*, *2μm* | This study |
| pPAR207 | *NUP84 S. mikatae* with *LEU2* and flanking sequence from the *NUP84 locus* | *URA3, LEU2*, *2μm* | This study |
| pPAR208 | *NUP84 S. bayanus* with *LEU2* and flanking sequence from the *NUP84 locus* | *URA3, LEU2*, *2μm* | This study |
| pPAR209 | *NUP84 S. kudriavzevii* with *LEU2* and flanking sequence from the *NUP84 locus* | *URA3, LEU2*, *2μm* | This study |
| pPAR211 | *NUP82 S. bayanus* with *KANMX6* and flanking sequence from the *NUP82 locus* | *URA3, KANMX6, 2μm* | This study |
| pPAR212 | *NUP82 S. kudriavzevii* with *KANMX6* and flanking sequence from the *NUP82 locus* | *URA3, KANMX6, 2μm* | This study |
| pPAR213 | *NUP82 S. mikatae* with *KANMX6* and flanking sequence from the *NUP82 locus* | *URA3, KANMX6, 2μm* | This study |
| pPAR240 | *URA3*-8opLexA-LacZ flanked by 1000bp of *ADE2* within pRS422 | *URA3, 2μm* | This study |
| pCMY-IT5 | *K. lactis URA3* flanked by two inverted SceI endonuclease recognition sites. | *K. lactis URA3* | C. Yellman |
| pGAL1-SCEK | SceI endonuclease under the control of the *GAL1* promoter. | *CEN, KANMX* | C. Yellman |

1. Yoshida K, Seo H-S, Debler EW, Blobel G, Hoelz A. Structural and functional analysis of an essential nucleoporin heterotrimer on the cytoplasmic face of the nuclear pore complex. Proc Natl Acad Sci U S A. 2011;108: 16571–16576. doi:10.1073/pnas.1112846108

2. Marshall KS, Zhang Z, Curran J, Derbyshire S, Mymryk JS. An improved genetic system for detection and analysis of protein nuclear import signals. BMC Mol Biol. 2007;8: 6. doi:10.1186/1471-2199-8-6

3. Curcio MJ, Garfinkel DJ. Single-step selection for Ty1 element retrotransposition. Proc Natl Acad Sci U S A. 1991;88: 936–940.

4. Dakshinamurthy A, Nyswaner KM, Farabaugh PJ, Garfinkel DJ. *BUD22* affects Ty1 retrotransposition and ribosome biogenesis in *Saccharomyces cerevisiae*. Genetics. 2010;185: 1193–1205. doi:10.1534/genetics.110.119115

5. Kaffman A, Rank NM, O'Shea EK. Phosphorylation regulates association of the transcription factor Pho4 with its import receptor Pse1/Kap121. Genes Dev. 1998;12: 2673–2683.
