# Supplementary material for: Control of yeast retrotransposons mediated through nucleoporin evolution
Source: PLoS Genet. 2018 Apr 25;14(4):e1007325. doi: 10.1371/journal.pgen.1007325 (PMC5918913; doi:10.1371/journal.pgen.1007325)
Supplement: S2 Table — A table containing the names and descriptions of all yeast strains used in this study and their origins. (DOCX) [file pgen.1007325.s008.docx]

**Table S2 – Yeast strains**

| **Strain** | **Genotype** | **Source** |
| --- | --- | --- |
| BY4741 | MATa *his3*Δ*1 leu2*Δ*0 met15*Δ*0 ura3*Δ*0* | [1] |
| BY4743 *nup82*Δ/*NUP82* | MATa/α *ura3*Δ*0 leu2*Δ*0 his3Δ1 LYS2+/lys2*Δ*0 met15*Δ*0/MET15+ can1*Δ*::LEU2+-MFA1pr-HIS3/CAN1+ nup82*Δ::*KANMX/NUP82* | SGA KO collection [2] |
| BY4741 *xrn1*Δ | MATa *his3*Δ*1 leu2*Δ*0 met15*Δ*0 ura3*Δ*0 xrn1*Δ | Haploid KO collection [3] |
| BY4741 *nup84*Δ | MATa *his3*Δ*1 leu2*Δ*0 met15*Δ*0 ura3*Δ*0 nup84*Δ | Haploid KO collection [3] |
| BY4741 *nup133*Δ | MATa *his3*Δ*1 leu2*Δ*0 met15*Δ*0 ura3*Δ*0 nup133*Δ | Haploid KO collection [3] |
| BY4741 *bud22*Δ | MATa *his3*Δ*1 leu2*Δ*0 met15*Δ*0 ura3*Δ*0 bud22*Δ | Haploid KO collection [3] |
| BY4741 *xrs2*Δ | MATa *his3*Δ*1 leu2*Δ*0 met15*Δ*0 ura3*Δ*0 xrs2*Δ | Haploid KO collection [3] |
| BY4741 *nup100*Δ | MATa *his3*Δ*1 leu2*Δ*0 met15*Δ*0 ura3*Δ*0 nup100*Δ | Haploid KO collection [3] |
| BY4741 *nup84*Δ | MATa *his3*Δ*1 leu2*Δ*0 met15*Δ*0 ura3*Δ*0 nup84*Δ | Haploid KO collection [3] |
| YPAR0130 | MATa *his3*Δ*1 leu2*Δ*0 met15*Δ*0 ura3*Δ*0 nup84*Δ::*NUP84-LEU2* (*S. cerevisiae*) | This study |
| YPAR0131 | MATa *his3*Δ*1 leu2*Δ*0 met15*Δ*0 ura3*Δ*0 nup84*Δ::*NUP84-LEU2* (*S. mikatae*) | This study |
| YPAR0132 | MATa *his3*Δ*1 leu2*Δ*0 met15*Δ*0 ura3*Δ*0 nup84*Δ::*NUP84-LEU2* (*S. bayanus*) | This study |
| YPAR0133 | MATa *his3*Δ*1 leu2*Δ*0 met15*Δ*0 ura3*Δ*0 nup84*Δ::*NUP84-LEU2* (*S. kudriavzevii*) | This study |
| YPAR0135 | MATa *his3*Δ*1 leu2*Δ*0 met15*Δ*0 ura3*Δ*0 nup84*Δ::*NUP84-LEU2* (*S. cerevisiae*) *ade2*::*URA3*::(LexAop)8-*LacZ* | This study |
| YPAR0137 | MATa *his3*Δ*1 leu2*Δ*0 met15*Δ*0 ura3*Δ*0 nup84*Δ::*NUP84-LEU2* (*S. mikatae*) *ade2*::*URA3*::(LexAop)8-*LacZ* | This study |
| YPAR0136 | MATa *his3*Δ*1 leu2*Δ*0 met15*Δ*0 ura3*Δ*0 nup84*Δ::*NUP84-LEU2* (*S. bayanus*) *ade2*::*URA3*::(LexAop)8-*LacZ* | This study |
| YPAR0138 | MATa *his3*Δ*1 leu2*Δ*0 met15*Δ*0 ura3*Δ*0 nup84*Δ::*NUP84-LEU2* (*S. kudriavzevii*) *ade2*::*URA3*::(LexAop)8-*LacZ* | This study |
| YPAR0139 | MATa *ura3*Δ*0 leu2*Δ*0 his3*Δ*1 can1*Δ::*LEU2+-MFA1pr-HIS3 nup82*Δ::*NUP82* (*S. cerevisiae*)*-KANMX6* | This study |
| YPAR0143 | MATa *ura3*Δ*0 leu2*Δ*0 his3*Δ*1 can1*Δ::*LEU2+-MFA1pr-HIS3 nup82*Δ::*NUP82* (*S. mikatae*)*-KANMX6* | This study |
| YPAR0141 | MATa *ura3*Δ*0 leu2*Δ*0 his3*Δ*1 can1*Δ::*LEU2+-MFA1pr-HIS3 nup82*Δ::*NUP82* (*S. kudriavzevii*)*-KANMX6* | This study |
| YPAR0142 | MATa *ura3*Δ*0 leu2*Δ*0 his3*Δ*1 can1*Δ::*LEU2+-MFA1pr-HIS3 nup82*Δ::*NUP82* (*S. bayanus*)*-KANMX6* | This study |
| YPAR0143 | MATa *ura3*Δ*0 leu2*Δ*0 his3*Δ*1 can1*Δ::*LEU2+-MFA1pr-his3*Δ::*HPHX6 nup82*Δ::*NUP82* (*S. cerevisiae*)*-KANMX6* | This study |
| YPAR0145 | MATa *ura3*Δ*0 leu2*Δ*0 his3*Δ*1 can1*Δ::*LEU2+-MFA1pr- his3*Δ::*HPHX6 nup82*Δ::*NUP82* (*S. mikatae*)*-KANMX6* | This study |
| YPAR0147 | MATa *ura3*Δ*0 leu2*Δ*0 his3*Δ*1 can1*Δ::*LEU2+-MFA1pr- his3*Δ::*HPHX6 nup82*Δ::*NUP82* (*S. kudriavzevii*)*-KANMX6* | This study |
| YPAR0149 | MATa *ura3*Δ*0 leu2*Δ*0 his3*Δ*1 can1*Δ::*LEU2+-MFA1pr- his3*Δ::*HPHX6 nup82*Δ::*NUP82* (*S. bayanus*)*-KANMX6* | This study |

1. Brachmann CB, Davies A, Cost GJ, Caputo E, Li JC, Hieter P, et al. Designer deletion strains derived from *Saccharomyces cerevisiae* S288C: a useful set of strains and plasmids for PCR-mediated gene disruption and other applications. Yeast. 1998;14: 115–132. doi:10.1002/(SICI)1097-0061(19980130)14:2<115::AID-YEA204>3.0.CO;2-2

2. Tong AHY. Systematic Genetic Analysis with Ordered Arrays of Yeast Deletion Mutants. Science. 2001;294: 2364–2368. doi:10.1126/science.1065810

3. Giaever G, Chu AM, Ni L, Connelly C, Riles L, Véronneau S, et al. Functional profiling of the *Saccharomyces cerevisiae* genome. Nature. 2002;418: 387–391. doi:10.1038/nature00935
